# DNA Ligase III Promotes Alternative Nonhomologous End-Joining during Chromosomal Translocation Formation

**DOI:** 10.1371/journal.pgen.1002080

**Published:** 2011-06-02

**Authors:** Deniz Simsek, Erika Brunet, Sunnie Yan-Wai Wong, Sachin Katyal, Yankun Gao, Peter J. McKinnon, Jacqueline Lou, Lei Zhang, James Li, Edward J. Rebar, Philip D. Gregory, Michael C. Holmes, Maria Jasin

**Affiliations:** 1 Developmental Biology Program, Memorial Sloan-Kettering Cancer Center, New York, New York, United States of America; 2 Weill Cornell Graduate School of Medical Sciences, New York, New York, United States of America; 3 Museum National d'Histoire Naturelle, Paris, France; 4 CNRS, UMR7196, Paris, France; 5 Inserm, U565, Paris, France; 6 Sangamo BioSciences, Richmond, California, United States of America; 7 Department of Genetics and Tumor Cell Biology, St Jude Children's Research Hospital, Memphis, Tennessee, United States of America; Brandeis University, United States of America

## Abstract

Nonhomologous end-joining (NHEJ) is the primary DNA repair pathway thought to underlie chromosomal translocations and other genomic rearrangements in somatic cells. The canonical NHEJ pathway, including DNA ligase IV (Lig4), suppresses genomic instability and chromosomal translocations, leading to the notion that a poorly defined, alternative NHEJ (alt-NHEJ) pathway generates these rearrangements. Here, we investigate the DNA ligase requirement of chromosomal translocation formation in mouse cells. Mammals have two other DNA ligases, Lig1 and Lig3, in addition to Lig4. As deletion of *Lig3* results in cellular lethality due to its requirement in mitochondria, we used recently developed cell lines deficient in nuclear Lig3 but rescued for mitochondrial DNA ligase activity. Further, zinc finger endonucleases were used to generate DNA breaks at endogenous loci to induce translocations. Unlike with Lig4 deficiency, which causes an increase in translocation frequency, translocations are reduced in frequency in the absence of Lig3. Residual translocations in Lig3-deficient cells do not show a bias toward use of pre-existing microhomology at the breakpoint junctions, unlike either wild-type or Lig4-deficient cells, consistent with the notion that alt-NHEJ is impaired with Lig3 loss. By contrast, Lig1 depletion in otherwise wild-type cells does not reduce translocations or affect microhomology use. However, translocations are further reduced in Lig3-deficient cells upon Lig1 knockdown, suggesting the existence of two alt-NHEJ pathways, one that is biased toward microhomology use and requires Lig3 and a back-up pathway which does not depend on microhomology and utilizes Lig1.

## Author Summary

Chromosomal rearrangements are associated with many tumor types, as they are one way in which genes affecting cancer initiation and progression become mutated. One type of rearrangement is a chromosomal translocation, in which parts of two different chromosomes join together. Although infrequent, translocations occur when both chromosomes undergo breakage and the ends from different chromosomes join rather than the two ends from the same chromosome. Human and mouse cells have three known DNA ligases which catalyze the joining of DNA ends (Lig1, Lig3, and Lig4). Lig4 is important for joining the correct ends together, thereby suppressing translocations. In this report, the role of the other two DNA ligases is examined in a novel mouse cell system. Lig3 is found to be required for efficient chromosomal translocation formation, but in its absence Lig1 can substitute, although less efficiently and although the joining characteristics of the two DNA ligases differ. These studies define the hierarchy of the three DNA ligases in this type of genomic rearrangement.

## Introduction

Recurrent reciprocal chromosomal translocations are hallmarks of several tumor types [1]. Breakpoint junction analysis indicates that translocations arise primarily through a nonhomologous end-joining (NHEJ) mechanism of double-strand break (DSB) repair in a process that results in a variety of DNA end modifications, including deletions and insertions. Notably, DNA ends frequently join at short sequence homologies of one or a few bases (microhomology) which may promote the joining reaction [2], [3].

A set of NHEJ factors has been defined based on their requirement both for cellular resistance to ionizing radiation and during V(D)J recombination for antigen receptor formation and diversity [4], [5]. These canonical NHEJ factors include the end protection protein Ku, DNA end processing enzymes, and the DNA ligase complex Lig4-XRCC4. Des pite the observation that translocation breakpoint junctions exhibit characteristics of NHEJ, the canonical pathway is not required for translocation formation; rather, this pathway is known to suppress translocations, as evidenced by the increased number of translocations arising in mouse cells deficient in components of this pathway. For example, canonical NHEJ deficiency in the context of p53 loss leads to pro-B cell lymphomas with Igh-Myc amplification and chromosomal translocation [6], [7]. Further, translocations involving induced DSBs on two different chromosomes are increased in frequency in either Ku or Lig4-XRCC4-deficient mouse embryonic stem (ES) cells, with breakpoint junctions showing similar end modifications and microhomology as in wild-type cells [8], [9], suggesting that canonical NHEJ does not play an important role in the joining events.

Although studies of NHEJ have focused on canonical NHEJ in the context of V(D)J recombination, the existence of an alternative pathway(s) of NHEJ has been evident from the earliest analyses of canonical NHEJ-deficient cells, using either plasmid or chromosomal substrates for DSB repair in rodent and human cells [10]–[16]. This alternative pathway, termed alt-NHEJ, is poorly defined, although recently several candidate components of this pathway have been proposed, including the Mre11 complex [17]–[19], the end resection protein CtIP [20]–[22], and poly (ADP-ribose) polymerases (PARPs) [23], [24]. The increase in translocations in canonical NHEJ-deficient mouse cells implies that alt-NHEJ is primarily responsible for their formation and, moreover, that alt-NHEJ leading to translocations is suppressed by the canonical pathway. Given that translocations appear to arise by alt-NHEJ even in the presence of the canonical pathway [9], they provide a good model with which to characterize components of the alt-NHEJ pathway.

As NHEJ ultimately involves DNA ligation and Lig4 is not required for translocation formation, one (or both) of the other two known DNA ligases present in mammalian cells, Lig1 and Lig3 [25], must be required for this process. Both of these ligases have essential cellular functions – the primary cellular role of Lig1 is for Okazaki fragment ligation during DNA replication [25], while Lig3 is essential for mitochondrial DNA metabolism [26], [27]. Lig3 interacts with the single-strand break repair protein XRCC1 via its C-terminal BRCT domain [28], [29]. Regarding DSB repair, Lig3 has been shown to have an end-joining activity in cell extracts [23] and in Lig4-deficient cells depleted for Lig3 using plasmid substrates, implicating Lig3 in a backup pathway of NHEJ [30].

Given that chromosomal translocations have been shown to arise by alt-NHEJ in mouse cells, we investigated the role of the three DNA ligases in this process. We demonstrate that in the absence of nuclear Lig3, translocations are reduced in frequency and that the residual translocation breakpoint junctions show less microhomology, demonstrating that Lig3 has a preference for joining ends at pre-existing microhomology. Lig3-dependent events do not require the C-terminal BRCT domain, indicating that interaction with XRCC1 is dispensable for these alt-NHEJ events. Knockdown of Lig1, but not Lig4, in the nuclear Lig3-deficient cells further reduces translocation formation, while having no effect in wild-type cells, indicating that it acts as a backup to Lig3 for these events. These experiments define Lig3 as having a primary role in this alt-NHEJ process even in the presence of canonical NHEJ and suggest the existence of multiple alt-NHEJ pathways.

## Results

### Chromosomal translocations are reduced in Lig3-deficient mouse cells

**Figure 1:**
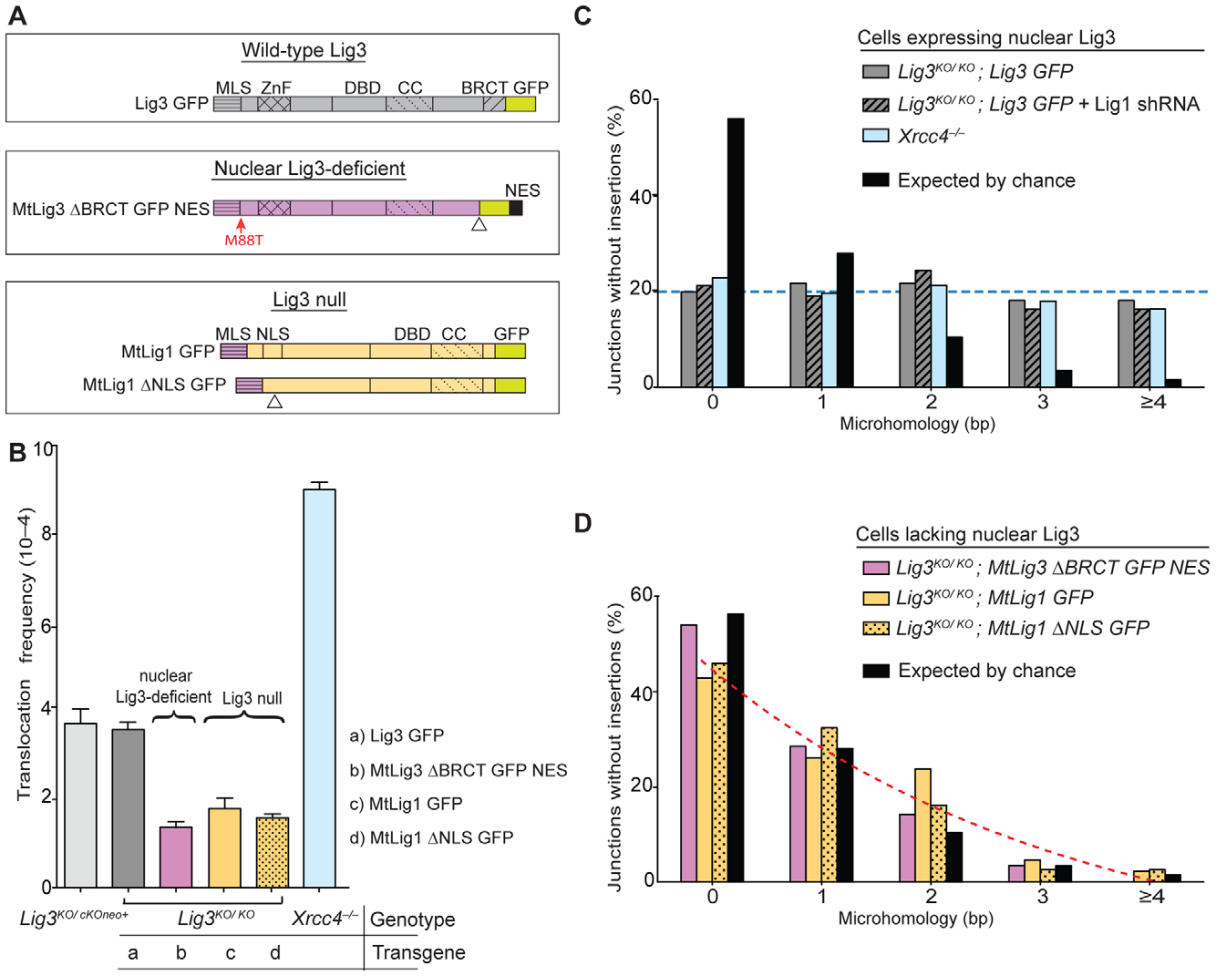
Lig3-deficient mouse cells have fewer chromosomal translocations and reduced microhomology at junctions. (A) DNA ligases expressed from transgenes in *Lig3^KO/KO^* cells. Wild-type Lig3 is expressed in control cells. Nuclear Lig3-deficient cells express mitochondria-restricted Lig3 (MtLig3-ΔBRCT-GFP-NES), while Lig3 null cells express mitochondria-targeted Lig1 (MtLig1 or MtLig1-ΔNLS). All transgenes are GFP tagged. MLS, mitochondrial leader sequence from Lig3 for mitochondrial localization; ZnF, zinc finger domain; BRCT, BRCA1 C-terminal related domain; DBD, DNA-binding domain; CC, catalytic core; *M88T, mutation of the Lig3 nuclear translation initiation site which disrupts translation of a nuclear-specific form of Lig3; NES, nuclear export signal derived from MAPKK to exclude Lig3 from the nucleus; ΔNLS, deletion of Lig1 nuclear localization signal (amino acids 135 to 147) to decrease nuclear localization of Lig1; ΔBRCT, deletion of theLig3 BRCT domain (amino acids 934 to 1009); GFP, green fluorescent protein. Triangles denote position of deletions of the indicated domains. A color scheme is used throughout the figures to assist in tracking various ligase forms (gray, wild-type Lig3; purple, mitochondria-only Lig3; orange, Lig1 forms). (B) Translocations are reduced in cell lines without nuclear Lig3. By contrast, translocations increase in cells deficient for Lig4–XRCC4 complex. (C) Microhomology length distributions for der(6) breakpoint junctions are similar for control cells, cells depleted for Lig1, and cells deficient for Lig4–XRCC4, and differ from that expected by the chance presence of microhomology. Only junctions with simple deletions (i.e., without an insertion) are included. The probability that a junction will have *X* nucleotides of microhomology by chance assumes an unbiased base composition and is calculated as previously described [9]. The transgenes present in the *Lig3^KO/KO^* cells are indicated in italics. (D) Microhomology length distributions in cells without nuclear Lig3 are shifted to a distribution similar to that expected by chance.

Lig3 is essential to cells [31] due to its ligase activity in mitochondria [26], [27]. We were able to rescue *Lig3^KO/KO^* mouse embryonic stem (ES) cells through pre-emptive complementation by expressing DNA ligases targeted to mitochondria [26]. In this approach, a *Lig3^KO/cKOneo+^* cell line, which contains one *Lig3* null allele and a second conditional allele with an intronic neomycin selection marker, was constructed (Figure S1). Transgenes expressing various DNA ligase forms fused to GFP were stably integrated into the *Lig3^KO/cKOneo+^* cells, which were then treated with Cre recombinase to transform the conditional *Lig3* allele to a second null allele. Cells specifically deficient for nuclear Lig3 or altogether deleted for Lig3 were constructed by this approach (Figure 1A) [26]. Viable Lig3 null cells were generated through expression of Lig1 fused to a mitochondrial leader sequence (MtLig1). MtLig1 was expressed at a fraction of the level of endogenous Lig1 in these cells [26], but to diminish the possibility that it contributes to nuclear ligation activity, we also deleted the Lig1 nuclear localization signal, generating MtLig1-ΔNLS. Additionally, nuclear Lig3-deficient cells were created by expressing a highly modified form of Lig3 (MtLig3-ΔBRCT-NES), where the nuclear translation initiation site was mutated, the BRCT domain implicated in nuclear transport [32] was deleted, and a potent nuclear export signal (NES) [33] was fused to the C-terminus. Lig3 null and nuclear Lig3-deficient cells are not sensitive to ionizing radiation [26], [27], suggesting that Lig3 is not required for global DSB repair, in contrast to the canonical NHEJ ligase, Lig4 [3].

**Figure 2:**
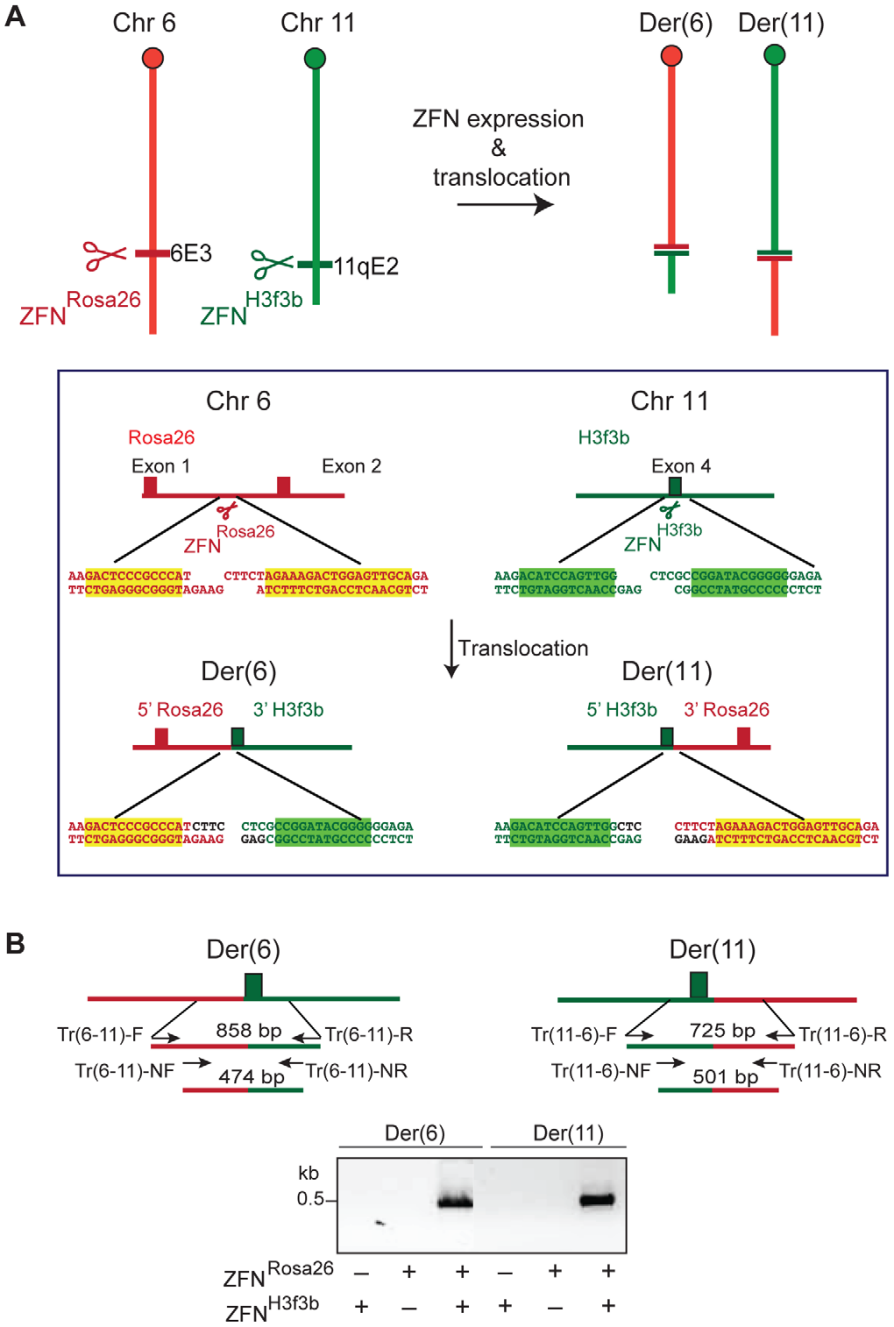
Induction of chromosomal translocations at endogenous mouse loci. (A) DSBs are induced at cleavage sites for the zinc finger nucleases (ZFNs) ZFN^Rosa26^ and ZFN^H3f3b^ on chromosomes 6 and 11, respectively. Joining of DNA ends from 2 different chromosomes can lead to the translocations generating derivative chromosomes der(6) and der(11). Sequences surrounding the DSBs generated by the ZFNs, together with sequences of the resultant translocation chromosomes, are shown. The latter assumes fill-in of the 5′ overhangs and no loss of sequence information from the DNA ends. (B) Nested PCR is used to identify translocation breakpoint junctions for der(6) and der(11), as shown, which is dependent on expression of both ZFN^Rosa26^ and ZFN^H3f3b^.

To address whether Lig3 plays a role in alt-NHEJ during translocation formation, DSBs were introduced at two loci in *Lig3^KO/KO^* rescued and parental cells at the *Rosa26* and *H3f3b* loci on chromosomes (chr) 6 and 11, respectively, by expressing zinc finger nucleases (ZFNs) [34] (Figure 2A and Figure S2). After allowing for translocation formation for 60 hours, translocation breakpoint junctions were amplified for both derivative chromosomes, der(6) and der(11), by nested PCR (Figure 2B), similar to an approach recently developed in human cells [35]. Using this approach, we quantified der(6) junctions in rescued and control cells and, for comparison, cells defective in the canonical NHEJ component XRCC4, the required Lig4 cofactor [25].

Translocations were readily detected in both parental cells and increased by a factor of 2.4 in *Xrcc4^−/−^* cells (3.7 vs 9×10^−4^; Figure 1B, Table 1), confirming previous results obtained with a different translocation system [9]. *Lig3^KO/KO^* cells rescued by wild-type Lig3 expression had a similar frequency of translocations as the parental cells expressing Lig3 from the endogenous locus. By contrast, translocations were substantially reduced in frequency in the absence of nuclear Lig3: translocations in both Lig3 null cells (*Lig3^KO/KO^* complemented with mitochondrial Lig1 transgenes) and nuclear Lig3-deficient cells (*Lig3^KO/KO^* complemented with the modified mitochondrial Lig3 transgene) were reduced by a factor of ∼2.3, ranging from 1.4 to 1.8×10^−4^ (Figure 1B, Table 1). Similar levels of intrachromosomal repair were observed in all cell lines, indicating that the reduced frequency of translocations was not due to reduced cleavage of the chromosomal loci (Figure S3A and S3B).

**Table 1:** Translocation frequencies for various cell lines tested.

		Translocation frequency (×10^−4^)	*P* value	
			a	b
*Lig3^KO/cKOneo+^*		3.7	0.5176	<0.0001
*Lig3^KO/KO^*	*MitLig3 ΔBRCT GFP NES*	1.4	<0.0001	
	*MitLig1 GFP*	1.8	0.0001	
	*MitLig1 ΔNLS GFP*	1.6	<0.0001	0.3662
	*Lig3 GFP*	3.6		
	*Lig3 ΔZNF GFP*	1.4	<0.0001	0.9439
	*Lig3 ΔBRCT GFP*	3.5	0.7622	<0.0001
*Xrcc4^−/−^*		9	<0.0001	<0.0001
*Lig3^KO/KO^*	*Lig3 GFP*+scr shRNA	3.7	0.6440	<0.0001
	*Lig3 GFP*+Lig1 shRNA	3.3	0.3528	<0.0001
	*Lig3 GFP*+Lig4 shRNA	7	0.0007	<0.0001
	*MitLig3 ΔBRCT GFP NES*+scr shRNA	1.4	<0.0001	0.8473
	*MitLig3 ΔBRCT GFP NES*+Lig1 shRNA	0.3	<0.0001	<0.0001
	*MitLig3 ΔBRCT GFP NES*+Lig4 shRNA	1.9	0.0011	0.1049

A two-tailed unpaired t-test was applied, with *P* values derived from a comparison with *Lig3^KO/KO^*; *Lig3 GFP* (a), and *Lig3^KO/KO^*; *MitLig3 ΔBRCT GFP NES (b)*.

### Translocation junctions demonstrate less microhomology in the absence of Lig3

**Figure 3:**
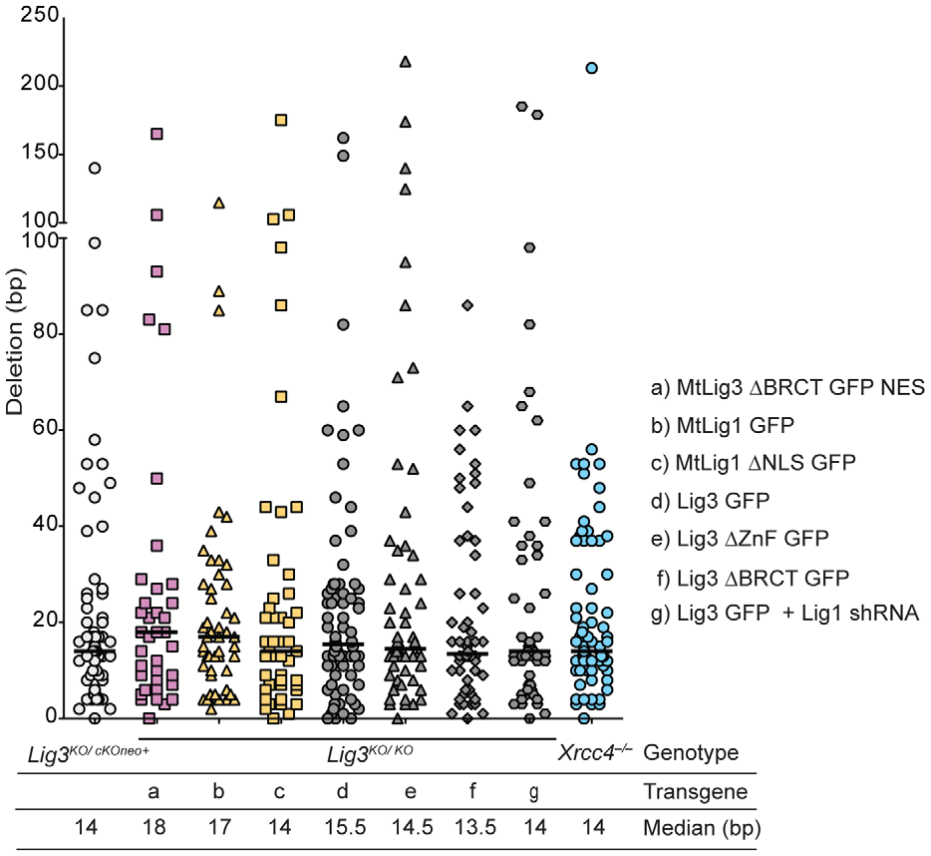
Deletion lengths for der(6) breakpoint junctions. Deletion lengths for the indicated genotypes do not differ significantly from each other. Each value represents the combined deletion from both ends of an individual junction. The median deletion length for each genotype is indicated by a bar on the graph and the value is given below the graph.

We next examined translocation breakpoint junctions. Similar microhomology, deletion, and insertion distributions were observed for cells expressing wild-type Lig3 and *Xrcc4^−/−^* cells (Figure 1C and Figure 3, Figures S4, S5, S6, Table 2), recapitulating what has been observed in another translocation system [9]. Notably, in both cell lines the microhomology distribution was different from that expected by chance (*p*<0.0001, two-tailed Mann-Whitney test; Figure S4), suggesting that microhomology drives many of these alt-NHEJ events between the two chromosomes. This contrasts with intrachromosomal joining which does not show a clear microhomology bias, except in the absence of the canonical NHEJ components [9],[14].

**Table 2:** Percent of translocation junctions without microhomology and with insertions.

		Translocation junctions		
		Without Microhomology	With Insertions	Total #
*Lig3^KO/cKOneo+^*		26%	18%	75
*Lig3^KO/KO^*	*MitLig3 ΔBRCT GFP NES*	55%	22%	37
	*MitLig1 GFP*	43%	18%	49
	*MitLig1 ΔNLS GFP*	48%	19%	47
	*Lig3 GFP*	21%	24%	74
	*Lig3 ΔZNF GFP*	16%	25%	59
	*Lig3 ΔBRCT GFP*	20%	19.6%	56
*Xrcc4^−/−^*		24%	18%	81
*Lig3^KO/KO^*	*Lig3 GFP*+scr shRNA	23%	27%	36
	*Lig3 GFP*+Lig1 shRNA	21%	15%	55
	*MitLig3 ΔBRCT GFP NES*+scr shRNA	45%	22%	40

By contrast, translocation junctions from Lig3 null and nuclear-deficient cell lines showed significantly reduced microhomology (compare red and blue dashed lines in Figure 1C and 1D). Notably, the microhomology distribution for junctions from each of the Lig3 null and nuclear Lig3-deficient cells was not significantly different from that expected by chance (Figure S4), indicating that pre-existing microhomology does not drive NHEJ events in the absence of Lig3. Thus, both the reduced frequency and the reduced microhomology at junctions demonstrate a role for Lig3 in alt-NHEJ leading to translocation formation. Unlike microhomology, no significant difference in the deletion and insertion distributions were observed (Figure 3, Figures S5 and S6, Table 2), suggesting that Lig3 does not affect the processing of the ends prior to ligation.

### Lig1, but not Lig4, is a backup DNA ligase for translocation formation

**Figure 4:**
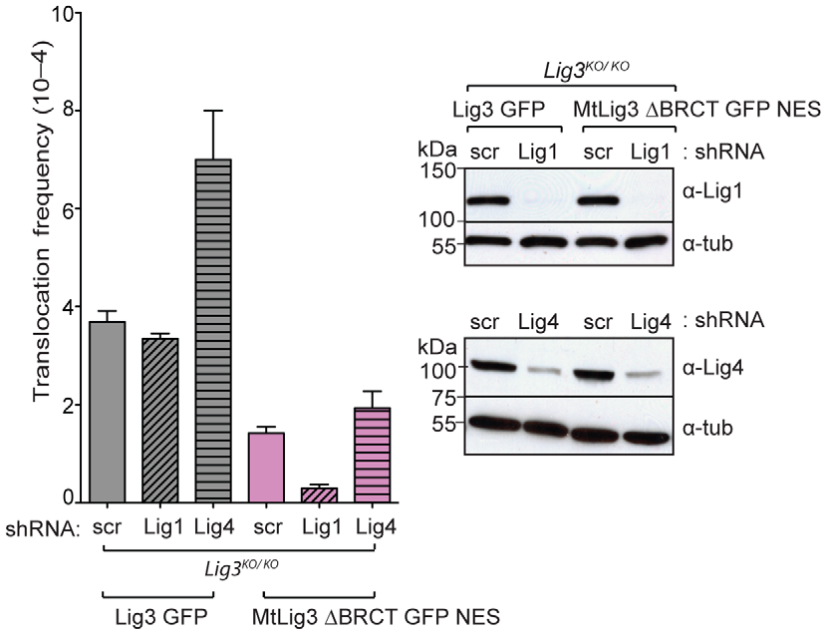
Lig1, but not Lig4, is a backup DNA ligase for translocation formation. Lig1 depletion results in reduced translocation frequency in nuclear Lig3-deficient cells (*Lig3^KO/KO^*; MtLig3 ΔBRCT GFP NES), but not in control cells expressing wild-type Lig3 (*Lig3^KO/KO^*; Lig3 GFP). By contrast, Lig4 depletion increases translocations significantly in wild-type cells but not nuclear Lig3-deficient cells. Lig1 and Lig4 lentiviral shRNA knockdown are similar in both nuclear Lig3-deficient cells and cells expressing wild-type Lig3. scr, shRNA with a scrambled sequence.

As translocations were not completely abolished in the absence of Lig3, we next addressed which of the other two DNA ligases was responsible for the remaining translocations. For this, we performed shRNA knockdowns for Lig1 or Lig4 (Figure 4). As expected, Lig4 depletion significantly increased translocations in cells expressing wild-type Lig3 (7×10^−4^; Figure 4, Table 1), similar to *Xrcc4^−/−^* cells (9×10^−4^; Figure 1B). By contrast, Lig4 depletion had little effect in nuclear Lig3-deficient cells (1.9 vs 1.4×10^−4^; Figure 4, Table 1), indicating that in the absence of Lig3 translocations did not occur by canonical NHEJ. While loss of Lig3 reduces translocations in otherwise wild-type cells (2.3-fold, ∼1.6 vs 3.7×10^−4^; Figure 4, Table 1), loss of Lig3 in Lig4-depleted cells leads to an even more severe decrease in translocations (3.7-fold, 1.9 vs 7×10^−4^, Table 1). These results reinforce the conclusion that Lig3 acts in the alt-NHEJ pathway to promote translocations whether or not Lig4 is present, whereas Lig4 acts in the canonical pathway to suppress translocations.

Next we examined the role of Lig1. Short-term depletion of Lig1 had no effect on survival of cells expressing wild-type Lig3, although survival was reduced about 20% in nuclear Lig3-deficient cells. Lig1 depletion in cells expressing wild-type Lig3 did not alter translocation frequency (3.3 vs 3.7×10^−4^; Figure 4, Table 1), indicating that Lig1 does not normally play a role in this alt-NHEJ pathway. Notably in nuclear Lig3-deficient cells, very few translocations were recovered upon Lig1 depletion. Thus, translocations were reduced by a factor of ∼12 in nuclear Lig3-deficient cells upon Lig1 depletion compared with cells expressing wild-type Lig3 (0.3 vs 3.7×10^−4^; Figure 4, Table 1). These results indicate that Lig1 can back-up Lig3 in alt-NHEJ for translocation formation, but that the participation of Lig1 is prominent only in the absence of Lig3.

Consistent with Lig1 having no effect on translocation frequency, we also observed that microhomology was not affected by Lig1 depletion in cells expressing wild-type Lig3 (*p* = 0.7859; Figure 1C, Figures S4 and S5). Given the low level of translocations, sufficient numbers of junctions could not be obtained for analysis from nuclear Lig3-deficient cells depleted for Lig1. Upon Lig1 depletion, similar levels of intrachromosomal repair were observed as in wild-type cells, indicating that the reduced frequency of translocations in nuclear Lig3-deficient cells was not due to reduced cleavage of the chromosomal loci (Figure S3C).

### Deletion of the ZnF domain of Lig3, but not the XRCC1-interacting BRCT domain, affects translocation frequency and outcome

**Figure 5:**
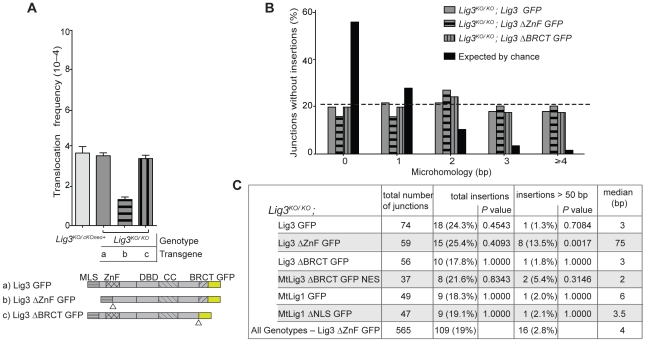
Analysis of Lig3 domain requirements in translocation formation. (A) Deletion of the Lig3 BRCT domain has no effect on translocation frequency, while deletion of the Lig3 ZnF domain reduces translocations. Triangles denote the deletions of the indicated domains. ΔZnF, deletion of Lig3 amino acids 89 to 258 including the ZnF itself and additional sequences which corresponds to a previously described Lig3 ΔZnF mutant [37], [38]; ΔBRCT, deletion of Lig3 amino acids 934 to 1009. (B) Microhomology length distributions in translocation junctions are similar in cells expressing wild-type Lig3 and those expressing either ZnF or BRCT deletions of Lig3. (C) Large, complex insertions are more frequent in cells expressing Lig3 deleted for the ZnF domain. The summary “All Genotypes – Lig3 ΔZnF” denotes all junctions analyzed except those from *Lig3^KO/KO^*; Lig3 ΔZnF cells. *P* values are calculated relative to this group with a Fisher's exact test.

Having established a role for Lig3 in alt-NHEJ, we next sought to determine which domains of Lig3 are important in this process. The BRCT domain of Lig3 interacts with the scaffold protein XRCC1 [28], which has been implicated in the recruitment of Lig3 to DNA damage foci [32]. When we delete this domain, the protein is still present in the nucleus, although at reduced levels (Figure S7) [32]. Lig3-ΔBRCT *Lig3^KO/KO^* cells were found to have indistinguishable translocation frequencies from cells expressing wild-type Lig3 (3.5 vs 3.6×10^−4^; Figure 5A, Table 1). Furthermore, translocation junctions showed very similar characteristics (Figure 3, Figures S5 and S6, Table 2), including microhomology (*p* = 0.9112; Figure 5B, Figure S4). Lig3 interaction with XRCC1, therefore, appears to be dispensable for alt-NHEJ leading to translocations.

Lig3 has an N-terminal zinc finger (ZnF) domain which interacts with PARP1 [36] and which has been reported to be critical for its intermolecular ligation activity *in vitro* [37], [38]. Lig3-ΔZnF *Lig3^KO/KO^* cells had a reduced translocation frequency compared with wild-type cells (1.4 vs 3.6×10^−4^; Figure 5A, Table 1). Microhomology in Lig3-ΔZnF *Lig3^KO/KO^* cells, as well as deletions, were not obviously different from wild-type cells (*p* = 0.3426; Figure 3 and Figure 5B, Figures S4 and S5, Table 2), in contrast to what was observed with nuclear Lig3-deficient cells. However, a significant fraction of the junctions were unique to the Lig3-ΔZnF *Lig3^KO/KO^* cells in that they had long insertions of >50 bp (13.5% vs 2.8% for all other genotypes) (Figure S6), such that the median insertion length was 75 bp compared with 4 bp for all other genotypes (Figure 5C). Taken together, these results suggest that the ZnF domain promotes efficient joining but is not required for microhomology use.

## Discussion

The lack of Lig3 in model organisms like yeast and the lack of Lig3 mutant mammalian cells had limited functional studies of Lig3 *in vivo*. The recent discovery that the cellular viability requirement for Lig3 depends on its role in mitochondria [26], [27] led to the development of cell lines that are deficient for Lig3 in the nucleus [26], allowing us to address the role of Lig3 in chromosomal translocation formation and alt-NHEJ. Using ZFNs, DSBs were introduced into endogenous loci in these cells without prior integration of reporter substrates; nested PCR allowed the recovery of chromosomal translocation junctions within 60 hours. This system works as efficiently in mouse ES cells as in human ES cells [35]. With this approach, we were able to systematically induce and analyze translocations in a variety of ligase deficient backgrounds in mouse cells. Given that they arise by alt-NHEJ even in the presence of the canonical NHEJ [9], translocations provide a good model with which to characterize components of the alt-NHEJ pathway.

Here, we establish that Lig3 is a component of the alt-NHEJ pathway leading to translocations in mouse cells, as Lig3 deficiency leads to a >2-fold decrease in translocation frequency. By examining an extensive number of translocation breakpoint junctions, we observed a redistribution of microhomology with Lig3 loss to that expected by chance, implying that Lig3 favors the use of microhomology during joining. Microhomologies are short, as would be expected from limited end resection which exposes single-strands for annealing [21] and from analysis of translocation junctions found in patients [2]. The use of short microhomologies in chromosomal rearrangements is further underscored by recent genome-wide analysis of breast cancers [39].

We also demonstrate that the role of Lig3 in translocation formation is independent of XRCC1, as deletion of the XRCC1-interacting BRCT domain does not affect either translocation frequency or breakpoint junction characteristics. Although Lig3 and XRCC1 have been suggested to work in a complex [25], our results are consistent with recent studies that have differentiated the roles of Lig3 and XRCC1 in DNA damage repair [26], [27]. In contrast, deletion of the ZnF domain results in a decrease in translocation frequency. The ZnF domain may promote intermolecular ligation in this context, joining two translocation partners, in agreement with a role for this domain in the ligation of oligomers *in vitro* [38]. The lack of a shift in microhomology use as seen in the absence of Lig3 argues that the ZnF domain is not required for microhomology use in translocations. However, an unusual class of junctions – those with long insertions (>50 bp) – was more prominent in cells expressing this protein. It is conceivable that the deletion of the ZnF domain results in slower kinetics of joining *in vivo*, which could allow for longer polymerization giving rise to insertions in a subset of junctions.

Consistent with previous results obtained in mouse cells [9], [14], [40], the loss of XRCC4 or Lig4 increases translocation formation, highlighting once again that the canonical NHEJ ligase suppresses translocations. Lig4–XRCC4 could act as a physical barrier together with Ku to prevent access of Lig3 to DNA ends for translocation formation; alternatively, this complex, together with other canonical NHEJ components, could promote the efficient joining of ends to narrow the kinetic window for translocation formation. Importantly, depletion of Lig4 in nuclear Lig3-deficient cells did not increase the translocation frequency as it did in wild-type cells, such that the relief of the translocation suppression by Lig4–XRCC4 is specifically related to Lig3 access to ends. Thus, Lig3 loss leads to an even greater fold decrease in translocation frequency in Lig4-depleted cells (3.7-fold) than it does in otherwise wild-type cells (2.3-fold). This suggests a more dominant role for Lig3 for translocation formation in the absence of the canonical NHEJ ligase.

**Figure 6:**
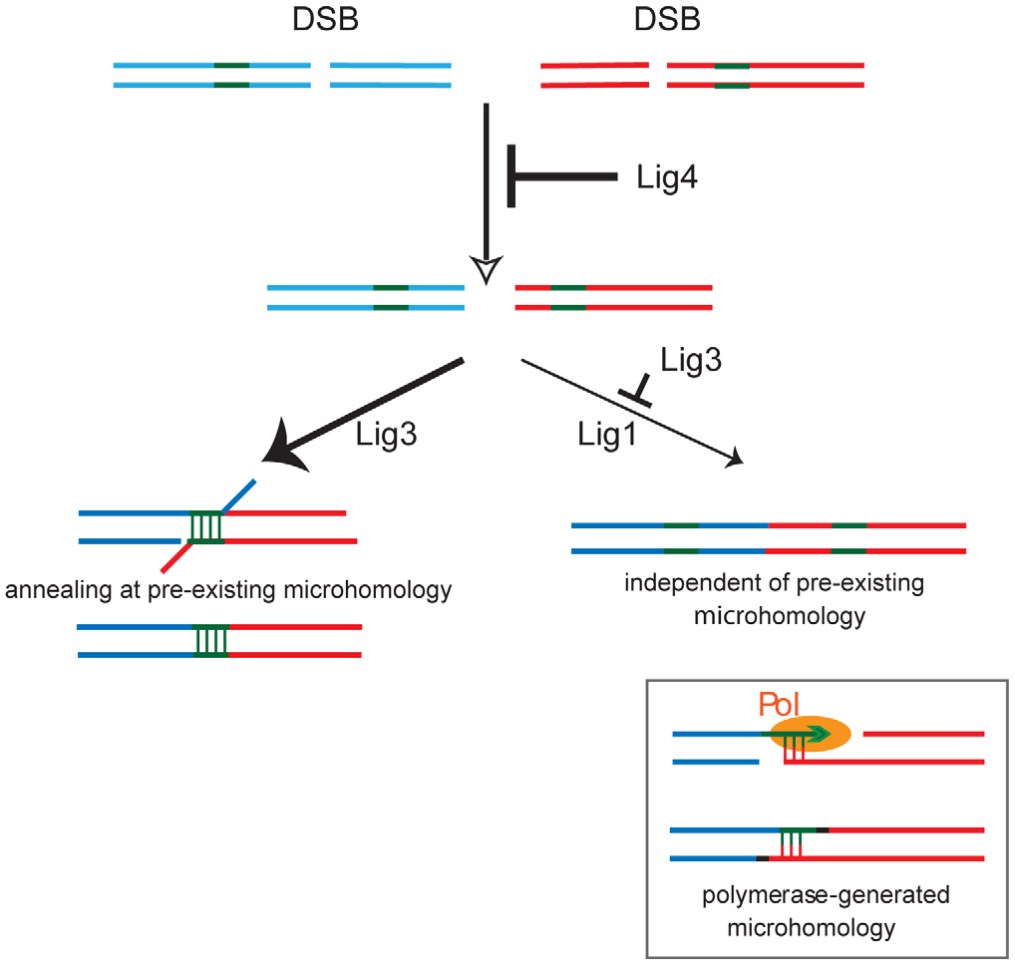
Model for the role of the three mammalian DNA ligases in chromosomal translocation formation in mouse cells. Upon DSB formation on two chromosomes (blue and red lines), Lig4 promotes intrachromosomal joining to maintain genomic integrity and suppresses translocations. Translocations are formed by Lig3, preferentially at short microhomologies (green lines). DNA end resection, which is likely suppressed by Lig4, forms single-stranded DNA to expose microhomologies which can anneal. In the absence of Lig3, Lig1 can form translocations independent of pre-existing microhomology. However, microhomology can also be generated by polymerase (Pol) activity at a DNA end.

We further found that Lig1 depletion in wild-type mouse cells does not have any effect on translocation frequency, whereas Lig1 depletion in nuclear Lig3-deficient cells nearly abolishes translocations. This implies that Lig3 has a primary role in alt-NHEJ resulting in translocations, but that Lig1 can function in the absence of Lig3 as a back-up ligase to provide limited activity, suggesting the existence of at least two alt-NHEJ pathways with these ligases operating in a hierarchy. Recent results examining base excision repair have also indicated that Lig1 and Lig3 can act in a hierarchy, although in this case Lig1 appears to be the primary ligase whereas Lig3 acts as the back-up ligase [27]. That these two ligases function in distinct pathways in translocations, as opposed to substituting for each other within one pathway, is supported by the different microhomology distributions: breakpoint junctions formed by Lig3 show a preference for pre-existing microhomology, whereas those formed by Lig1 do not (Figure 6). We cannot exclude that microhomology is generated by short polymerization (polymerase-generated microhomology) [3] that would promote Lig1-dependent joining. Polymerase-generated microhomology could also account for the 0 bp microhomology class of joining events that occur in the presence on Lig3; alternatively, there may not be a strict dependence on microhomology for joining by Lig3. The short polymerization may be template-dependent (Figure 6) or arise by chance in a template-independent manner (not shown). Although polymerase-generated microhomology cannot be scored, the existence of short insertions at translocation breakpoint junctions (either template dependent or independent) provides evidence for polymerization at DNA ends [9].

In the end, NHEJ is a DNA ligation process [41], and here we establish the intricate interplay of the three DNA ligases in alt-NHEJ leading to translocations in mouse cells. Lig3 promotes alt-NHEJ, but in its absence Lig1 can also function in this process. The roles for Lig3 and Lig1 contrast with that of Lig4, which suppresses chromosomal translocations.

## Materials and Methods

### Western blotting

Whole cell extracts were prepared with Nonidet-P40 buffer and were run on a 7.5% (w/v) Tris-HCl SDS page gel, blotted, and then probed with Lig3 antibody clone 7 (BD Transduction Labs), which recognizes both the human and mouse Lig3 proteins, or Lig1 antibody N-13 (Santa Cruz). α-tubulin (Sigma) was used as a loading control.

### Translocation analysis

The ZFN^Rosa26^ pair (Sigma-Aldrich) was designed and tested as described using an archive of validated 2-finger modules [42], [43]. ZFN^Rosa26^ was obtained from Sigma-Aldrich; ZFN^H3f3b^ was previously described [44]. For translocation frequency analysis, a similar approach to the method recently described in human cells was used [35]. Basically, for transfection with ZFN plasmids, 1×10^6^ ES cells were plated in each well of a 6-well plate and 4 hours later, the ZFN^Rosa26^ pair (0.5 µg 18473 and 2.5 µg 18477 plasmids) and ZFN^H3f3b^ pair (0.5 µg 25000 and 0.5 µg 25001) were transfected with Lipofectamine 2000 (Invitrogen), according to manufacturer's instructions. After 6 hours, cells were plated in a 96-well plate at a density of 2×10^4^ cells per plate. For translocation analysis, cells were lysed 60 hours after transfection directly in the 96-well plate in 40 µl lysis buffer (10 mM Tris pH 8.0, 0.45% (v/v) Nonidet P-40, 0.45% (v/v) Tween20) per well. The lysate was incubated with 100 µg/ml Proteinase K at 55°C for 2 hours and then incubated at 95°C for 10 min before PCR. The first round of PCR primers used for der(11) are: Tr(11-6)- F 5′-TTGACGCCTTCCTTCTTCTG-3′ and Tr(11-6)- R 5′- GCACGTTTCCGACTTGAGTT -3′, used at an annealing temperature of 62°C. The second round, nested PCR primers are: Tr(11-6)- NF 5′- CTGCCATTCCAGAGATTGGT -3′ and Tr(11-6)- NF 5′- TCCCAAAGTCGCTCTGAGTT -3′, used at an annealing temperature of 62°C. For PCR quantification of der(6) translocation frequency, the primers indicated in Figure 2 were: Tr(6-11)- F 5′-GCGGGAGAAATGGATATGAA-3′, Tr(6-11)- R 5′-AACCTTTGAAAAAGCCCACA-3′, Tr(6-11)- NF 5′-GGCGGATCACAAGCAATAAT-3′, and Tr(6-11)- NR 5′-AGCCACAGTGCTCACATCAC-3′. The first round of PCR was performed with 7 µl cell lysate from each well in a total of 50 µl per well (24 cycles, annealing temperature of 60°C). Then, 0.5 µl of the first PCR was used in a second, nested PCR (40 cycles, annealing temperature of 60°C) with SYBR Green for qPCR (Stratagene MX3005). The PCR cycle contains a denaturation curve cycle. Nested PCR fragments corresponding to translocation junctions are ∼503 bp, and have melting temperature between 87–90°C. For each experiment, cells were counted at the time of lysis to ensure that there was no growth perturbation in the experiment. For breakpoint sequence analysis, PCR reactions positive for translocation formation were purified using a PCR purification kit (Invitrogen) and sent for sequencing with Tr(6-11)-1NF and Tr(6-11)-1NR primers.

### Lentiviral Lig1 and Lig4 knock down

Purified pLKO.1-puro plasmids containing shRNA (Sigma) were transfected into 293T cells, using Mission Lentiviral packaging mix (Sigma, SHP001) and Fugene 6 (Roche). Infectious lentiviruses were harvested at 48 hours posttransfection and filtered through a 0.45 µm filter. 4×10^4^ ES-cells per well were seeded in 12-well plates with medium containing 4 µg/ml polybrene (Sigma). After 16 hours, cells were incubated with 750 µl of lentiviral particles with 4 µg/ml polybrene. After 4 hours, 750 µl of ES cell medium was added. After 24 hours, medium was changed with 1.6 µg/ml puromycin (Sigma) and after 3.5 days in puromycin selection cells are plated for translocation analysis.

### Surveyor nuclease assay

Surveyor nuclease assay was performed as previously described [42]. Basically, 1×10^6^ ES-cells transfected with none or both of the ZFN^Rosa26^ and ZFN^H3f3b^ were used as templates to amplify either Rosa26 or H3f3b regions. These samples were further used to quantify translocations. The Rosa26 or H3f3b region was amplified in the presence of ^32^P labelled dNTPs using AccuPrime taq DNA polymerase (Invitrogen) with the following primers, respectively: Rosa26FW1 5′- TAAAACTCGGGTGAGCATGT -3′ and RosaRv1 5′- GGAGTTCTCTGCTGCCTCCTG -3′ with an annealing temperature of 61°C; H3f3bFw 5′- GCGGCGGCTTGATTGCTCCAG -3′ and H3Rv1 5′- AGCAACTTGTCACTCCTGAGCCAC -3′ with an annealing temperature of 61°C. 2 µL of each PCR was mixed with 1× Accuprime buffer II and incubated as follows: 95°C for 5 min, 95–85°C at −2°C/s, 85–25°C at −0.1°C/s; hold at 4°C. This step melts and randomly reanneals the amplicons, which converts any mutations into mismatched duplex DNA. 1 µL Surveyor nuclease (Transgenomic) was incubated for 20 min at 42°C and the sample was run in a 10% acrylamide (BioRad). The cleaved bands were quantified by ImageQuant 5.1. Imprecise NHEJ percent is calculated using the formula: % indel = 100×(1−(1−fraction cleaved)^1/2^).

### Single-break NHEJ by bacterial colony hybridization

Mouse ES cells transfected with none or both of the ZFN^Rosa26^ and ZFN^H3f3b^ were used as templates to amplify either the Rosa26 or H3f3b region. The same primers used in the Surveyor nuclease assay were used. Amplicons were cloned by TOPO-TA (Invitrogen) and transformed into bacteria, similar to an approach recently described [35]. The probes used to determine precise NHEJ for Rosa26 or H3f3b loci are as follows, respectively: ProbeRosa26Nor- 5′- CGCCCATCTTCTAGAAAG-3′; ProbeH3Nor- 5′-CCAGTTGGCTCGCCGGAT-3′.

## Supporting Information

Figure S1 Pre-emptive complementation strategy for deletion of the endogenous *Lig3* gene in mouse embryonic stem (ES) cells. A *Lig3^KO/cKOneo+^* cell line was constructed which contains one *Lig3* null allele and a second conditional allele with an intronic neomycin selection marker. Transgenes expressing various DNA ligase cDNAs were stably integrated into the *Lig3^KO/cKOneo+^* cells, which were then treated with Cre recombinase to transform the conditional *Lig3* allele to a second null allele [26]. *Lig3^KO/KO^* clones were identified by their lack of growth in G418 (*neo−*). KO, knockout; cKOneo+, conditional knockout allele containing a functional *neo* gene.
(PDF)

Figure S2 Western blotting demonstrates that ZFN pairs are expressed at similar levels in the parental *Lig3^KO/cKOneo+^* cells and *Lig3^KO/KO^* cells expressing a DNA ligase transgene.
(PDF)

Figure S3 Analysis of imprecise intrachromosomal NHEJ at the ZFN^Rosa26^ and ZFN^H3f3b^ loci. A) Surveyor nuclease assay for Lig3 null and nuclear Lig3-deficient cells. Genomic DNA from ES cells transfected with no ZFN or both ZFN^Rosa26^ and ZFN^H3f3b^ that was used to quantify translocation frequency is also used as a template to amplify across the cleavage site at either the Rosa26 or H3f3b locus to quantify intrachromosomal NHEJ. After PCR, the amplification products are denatured and then reannealed to form heteroduplexes between unmodified and modified sequences from imprecise NHEJ. The mismatched duplex is selectively cleaved by Surveyor nuclease at the loops that form at the site of the mismatch. The percentage of locus modification from insertion/deletion (% Indel) is indicated at the bottom of each sample and provides an estimate imprecise of NHEJ at the Rosa26 and H3f3b loci. Given that the assay is sensitive to 1% locus modification, similar levels of imprecise NHEJ are observed for all cell lines tested. B) Analysis of intrachromosomal NHEJ by bacterial colony hybridization. The amplified product from (A) is also cloned using the TOPO-TA cloning system and transformed into bacteria. Bacterial colonies are hybridized with probes for unmodified Rosa26 or H3f3b ZFN target sequences. Colonies that hybridize with either of these probes will be from unmodified loci, while those that do not are from modified loci arising from imprecise NHEJ. Therefore, percent imprecise NHEJ (% Indel) at each locus is calculated as ratio of the number of colonies that do not hybridize with either of these probes to the total number of colonies analyzed. Plasmids from colonies that do not hybridize are sequenced to confirm that they contain imprecise NHEJ events. The imprecise NHEJ frequency obtained with this method is similar to the Surveyor nuclease assay results. C) Surveyor nuclease assay for the Lig1 depletion experiments. Similar levels of imprecise NHEJ were observed in the nuclear Lig3-deficient cells with or without Lig1 depletion, indicating that the greatly reduced frequency of translocations was not due to reduced cleavage of the chromosomal loci.
(PDF)

Figure S4 Statistics for microhomology distribution. A two-tailed Mann-Whitney test was applied, with *P* values derived from a comparison with *Lig3^KO/KO^*; *Lig3 GFP* (a) and Expected by chance (b).
(PDF)

Figure S5 Der(6) translocation junction sequences obtained from the indicated genotypes. ZFN recognition sites are indicated in red (chr. 6) and blue (chr. 11), with fill-in of the 5′ overhang for chr. 6. The cleavage site for chr. 11 is likely to be variable in whether it includes the terminal G (in parentheses) or not; therefore, the terminal G was not scored as an insertion in the junctions. Sequences are annotated as follows: del, deletion length from the DNA end after DSB formation; underline, microhomology; middle black letters, sequence of short insertion; +, length of long insertion. If the deletion extends beyond the chromosome sequence indicated at the top of the figure, a few bp flanking the deletion are indicated, with microhomology underlined.
(PDF)

Figure S6 Derivation of insertions found for der(6) breakpoint junctions. For each translocation breakpoint junction, deletion lengths from the chromosomes 6 and 11 ends are indicated in red and green boxes on the far left and far right, respectively, with the inserted segments represented as elevated boxes connected to each end. Inserts derived from chromosomes 6 and 11 are indicated in the elevated red and green boxes, respectively, while those derived from unknown sources are in white boxes. Included in this analysis are all inserted sequences >6 bp. An accession number is provided for an insert derived from another chromosome. Microhomologies are boxed. Inv, insertion is inverted.
(PDF)

Figure S7 Plasmids expressing the various GFP-tagged DNA ligases were transiently transfected into mouse ES cells and imaged to visualize localization of the protein. Mitochondria and the nucleus were labeled with Mitotracker Red CMXRos (Invitrogen) and Hoechst 33342 (Invitrogen), respectively. Wild-type Lig3-GFP is found in the nucleus and mitochondria. With the deletion of the BRCT domain of Lig3, levels in the nucleus decrease, although Lig3-ΔBRCT-GFP is still readily detected in the nucleus.
(PDF)
