# Supplementary figures and images for: DNA Ligase III Promotes Alternative Nonhomologous End-Joining during Chromosomal Translocation Formation
Source: PLoS Genet. 2011 Jun 2;7(6):e1002080. doi: 10.1371/journal.pgen.1002080 (PMC3107202; doi:10.1371/journal.pgen.1002080)

Figure S1

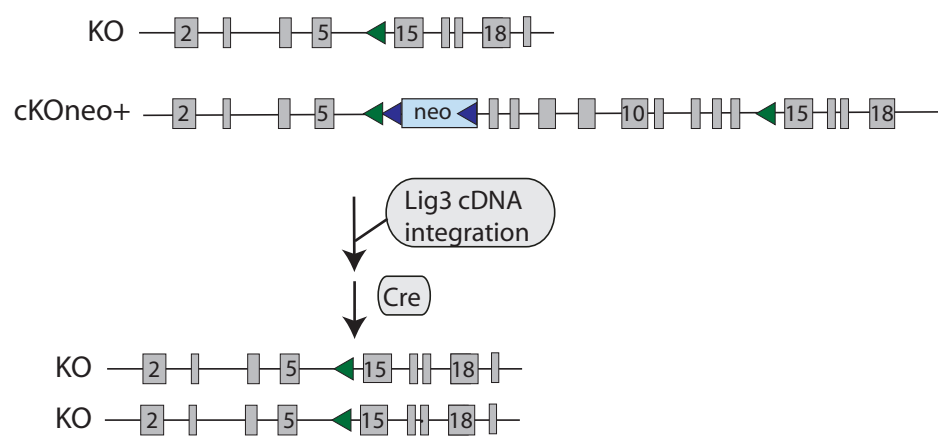

Supplement: Figure S1 — Pre-emptive complementation strategy for deletion of the endogenous Lig3 gene in mouse embryonic stem (ES) cells. A Lig3KO/cKOneo+ cell line was constructed which contains one Lig3 null allele and a second conditional allele with an intronic neomycin selection marker. Transgenes expressing various DNA ligase cDNAs were stably integrated into the Lig3KO/cKOneo+ cells, which were then treated with Cre recombinase to transform the conditional Lig3 allele to a second null allele [26]. Lig3KO/KO clones were identified by their lack of growth in G418 (neo−). KO, knockout; cKOneo+, conditional knockout allele containing a functional neo gene. (PDF) [file pgen.1002080.s001.pdf]

Figure S2

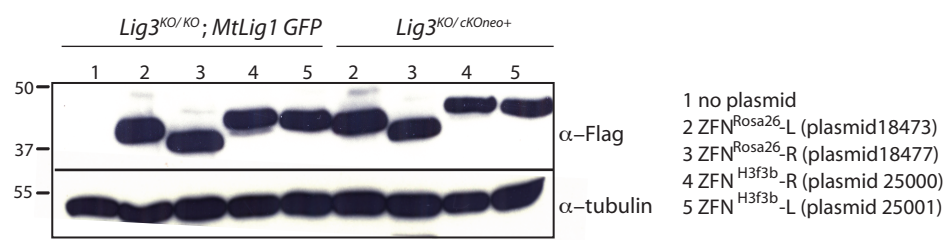

Supplement: Figure S2 — Western blotting demonstrates that ZFN pairs are expressed at similar levels in the parental Lig3KO/cKOneo+ cells and Lig3KO/KO cells expressing a DNA ligase transgene. (PDF) [file pgen.1002080.s002.pdf]

Figure S6

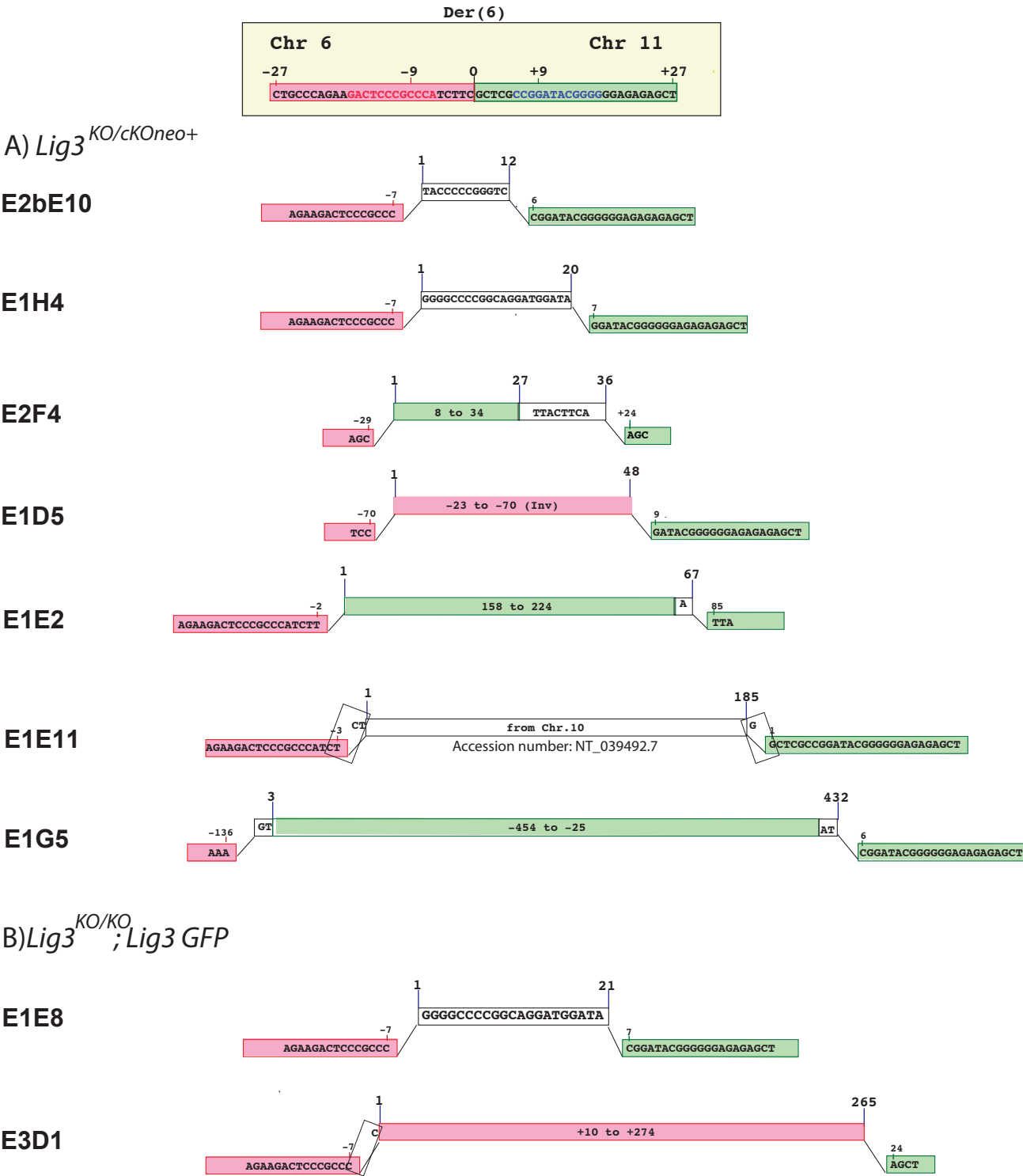

Figure S6

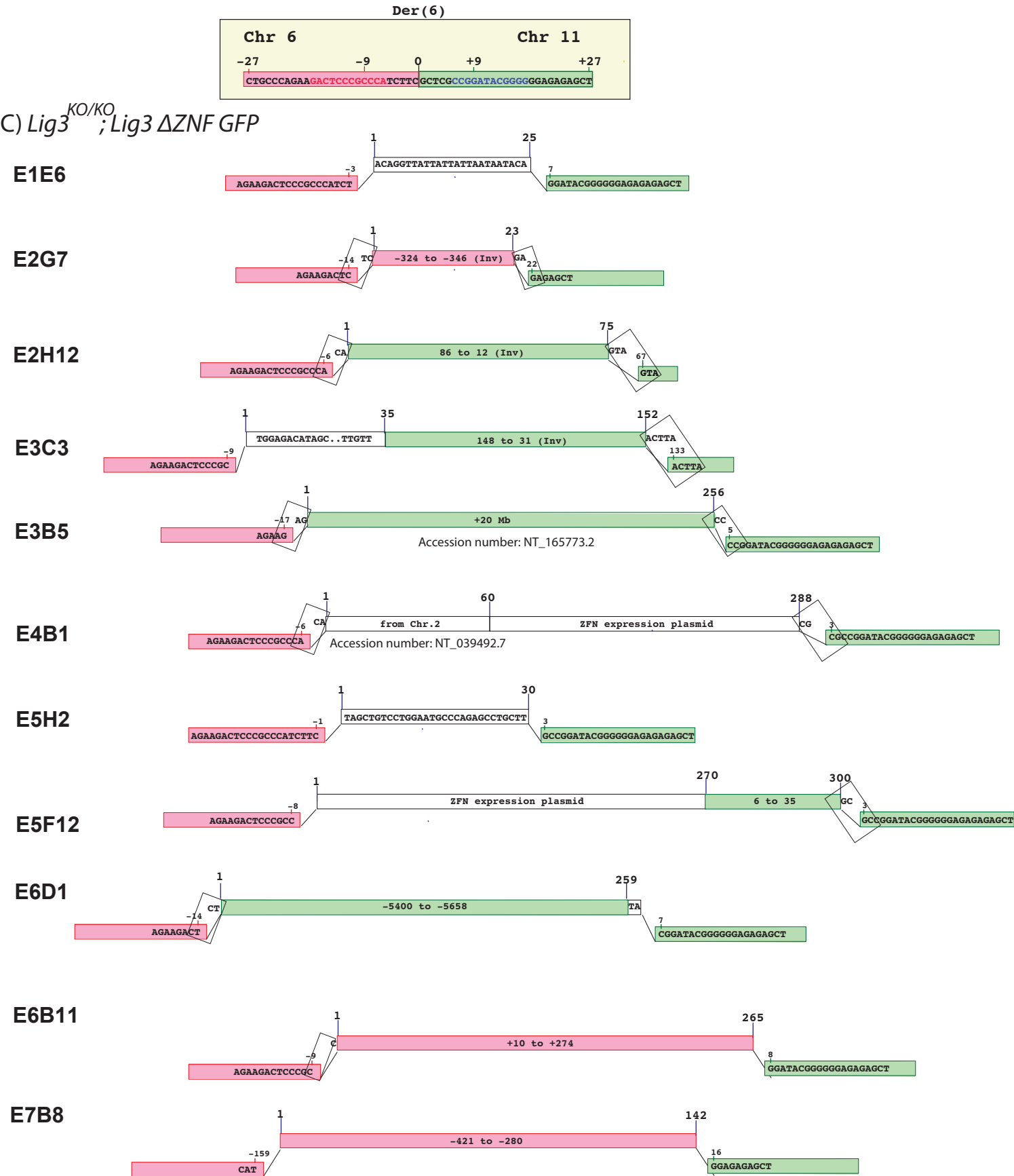

Figure S6

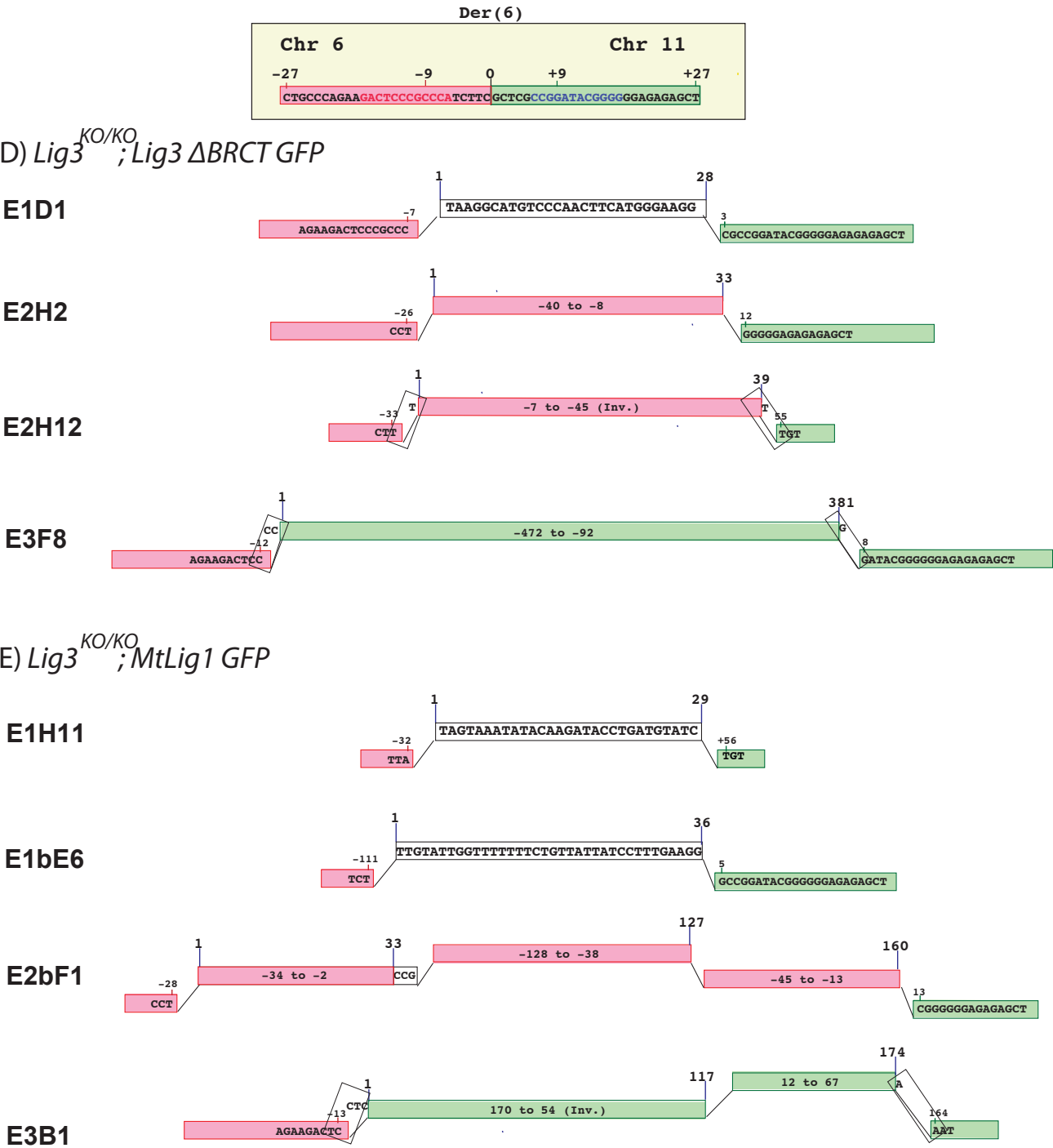

Figure S6

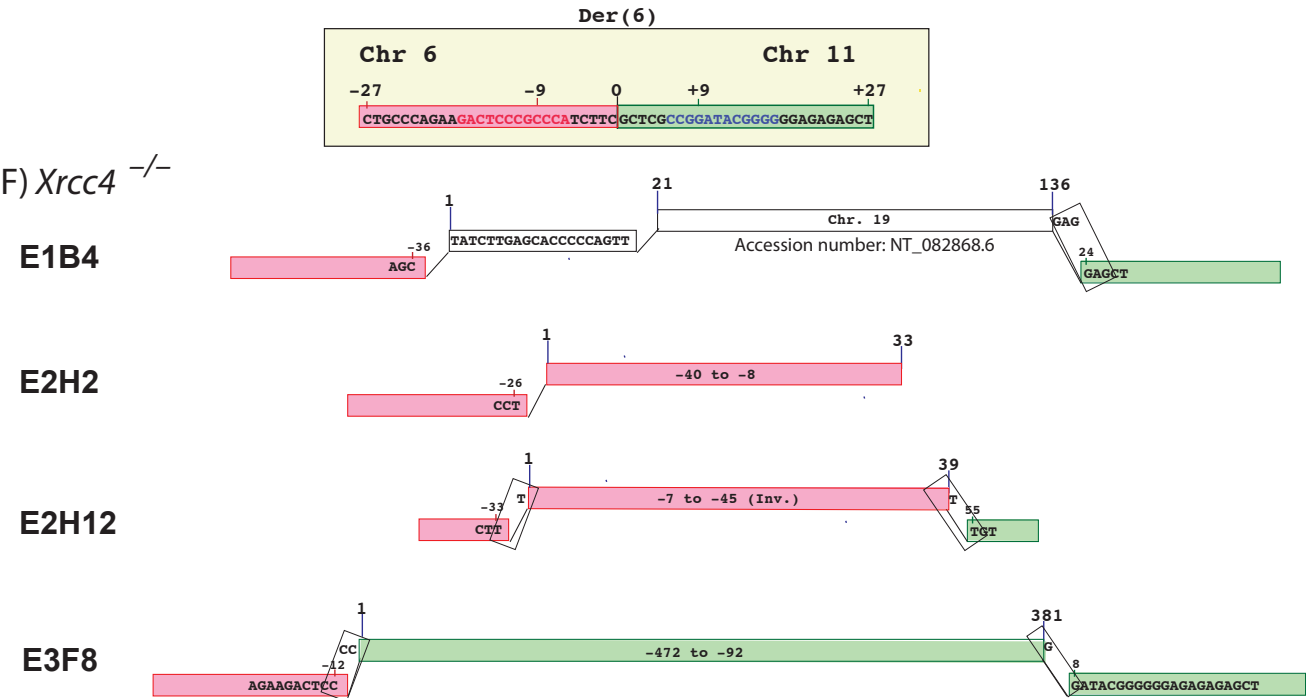

Supplement: Figure S6 — Derivation of insertions found for der(6) breakpoint junctions. For each translocation breakpoint junction, deletion lengths from the chromosomes 6 and 11 ends are indicated in red and green boxes on the far left and far right, respectively, with the inserted segments represented as elevated boxes connected to each end. Inserts derived from chromosomes 6 and 11 are indicated in the elevated red and green boxes, respectively, while those derived from unknown sources are in white boxes. Included in this analysis are all inserted sequences >6 bp. An accession number is provided for an insert derived from another chromosome. Microhomologies are boxed. Inv, insertion is inverted. (PDF) [file pgen.1002080.s006.pdf]

Figure S7

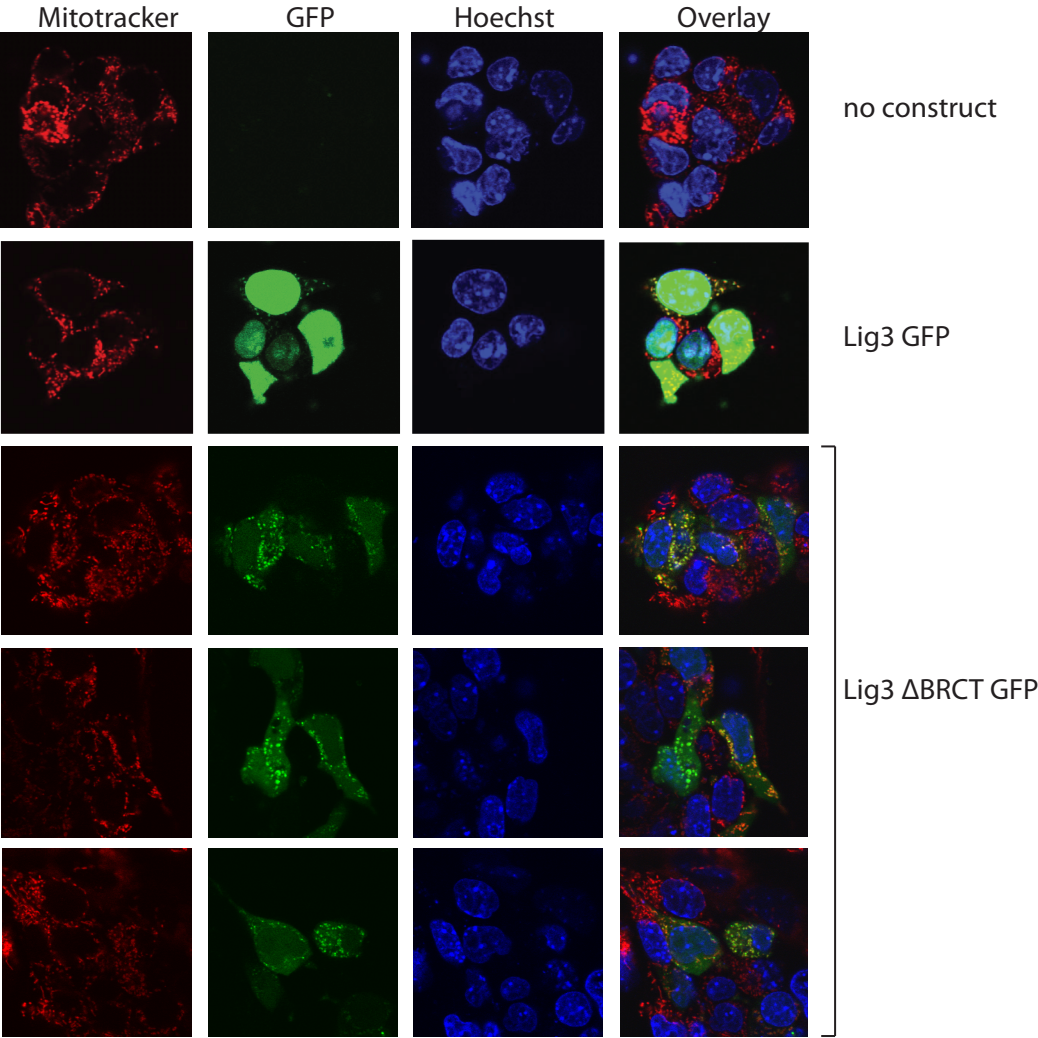

Supplement: Figure S7 — Plasmids expressing the various GFP-tagged DNA ligases were transiently transfected into mouse ES cells and imaged to visualize localization of the protein. Mitochondria and the nucleus were labeled with Mitotracker Red CMXRos (Invitrogen) and Hoechst 33342 (Invitrogen), respectively. Wild-type Lig3-GFP is found in the nucleus and mitochondria. With the deletion of the BRCT domain of Lig3, levels in the nucleus decrease, although Lig3-ΔBRCT-GFP is still readily detected in the nucleus. (PDF) [file pgen.1002080.s007.pdf]
