# Supplementary material for: DNA Ligase III Promotes Alternative Nonhomologous End-Joining during Chromosomal Translocation Formation
Source: PLoS Genet. 2011 Jun 2;7(6):e1002080. doi: 10.1371/journal.pgen.1002080 (PMC3107202; doi:10.1371/journal.pgen.1002080)

Figure S3

A) Surveyor nuclease assay for Lig3 null and nuclear Lig3-deficient cells

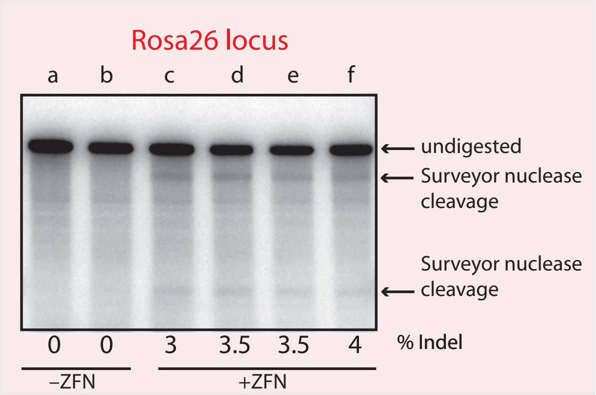

Lig3$^{KO/KO}$;
a) Lig3 GFP
b) MtLig1 ΔNLS GFP
c) *Lig3$^{KO/cKOneo+}$*
Lig3$^{KO/KO}$;
d) Lig3 GFP
e) MtLig1 ΔNLS GFP
f) MtLig3 ΔBRCT GFP NES

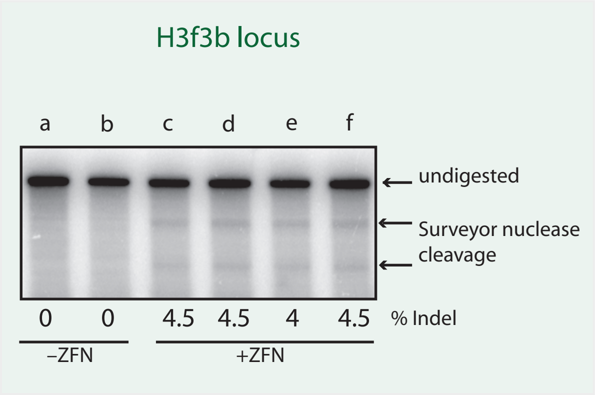

B) Bacterial colony hybridization for Lig3 null and nuclear Lig3-deficient cells

Lig3$^{KO/KO}$;	% Indel: Rosa26 locus	% Indel: H3f3b locus
Lig3 GFP	6/117 = 5.1 %	2/64 = 3 %
MtLig3 ΔBRCT GFP NES	4/90 = 4.4 %	5/113 = 4.4 %

Lig3$^{KO/KO}$; Lig3 GFP (Rosa26)

AAGACTCCCGCCCAT CTTCTAGAAAGACTGGAGTTGCAGA

del 133 TCTAGAAAGACTGGAGTTGCAGA
AAGACTCCCGCCCAT CTAGAAAGACTGGAGTTGCAGA
AAGACTCCCGCCCA GAAAGACTGGAGTTGCAGA
AAGACTCCCGCCCAT CTTTCAGAAAGACTGGAGTTGCAGA
AAGACT GGAGTTGCAGA
AAGACTCCCGCCCATCT AGAAAGACTGGAGTTGCAGA

Lig3$^{KO/KO}$; MtLig3 ΔBRCT GFP NES (Rosa26)

AAGACTCCCGCCCAT CTTCTAGAAAGACTGGAGTTGCAGA

AAGACTCCCGCCCAT TTTT TAGAAAGACTGGAGTTGCAGA
del 122 CAGA
AAGACTCCCGCCCAT AGAAAGACTGGAGTTGCAGA
AAGACTCCCG AAAGACTGGAGTTGCAGA

Lig3$^{KO/KO}$; Lig3 GFP (H3f3b)

GCCCAAAGACATCCAGTTGG CTCGCCGGATACGGGGGGAGAGA

GCCCAAAGACATC GGGGGGAGAGA
GCCCAAAGACATCCAG ATA CGGATACGGGGGGAGAGA

Lig3$^{KO/KO}$; MtLig3 ΔBRCT GFP NES (H3f3b)

GCCCAAAGACATCCAGTTGG CTCGCCGGATACGGGGGGAGAGA

G GGGGTGA GATACGGGGGGAGAGA
GCCCAAAGACATCCAGTTGGCTCGCTCGCCGGATACGGGGGGAGAGA
GCCCA CCGGATACGGGGGGAGAGA
GCCCAAAGACATCCAGTTG CTCGCCGGATACGGGGGGAGAGA
GCCCAAAGACATC GCCGGATACGGGGGGAGAGA

Figure S3

C) Surveyor nuclease assay for Lig1 knock down

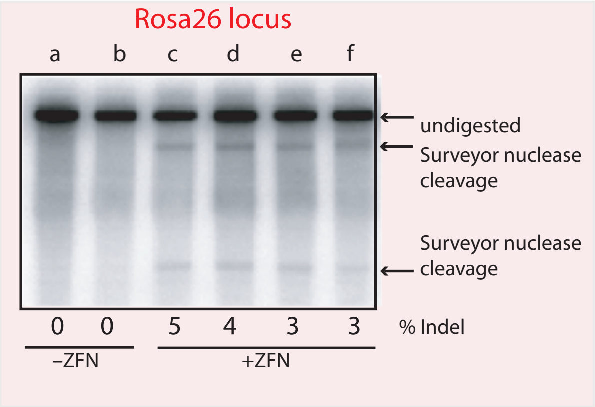

Lig3$^{KO/KO}$;

a) Lig3 GFP

b) MtLig3 ΔBRCT GFP NES

c) Lig3 GFP +scr shRNA

d) Lig3 GFP +Lig1 shRNA

e) MtLig3 ΔBRCT GFP NES +scr shRNA

f) MtLig3 ΔBRCT GFP NES +Lig1 shRNA

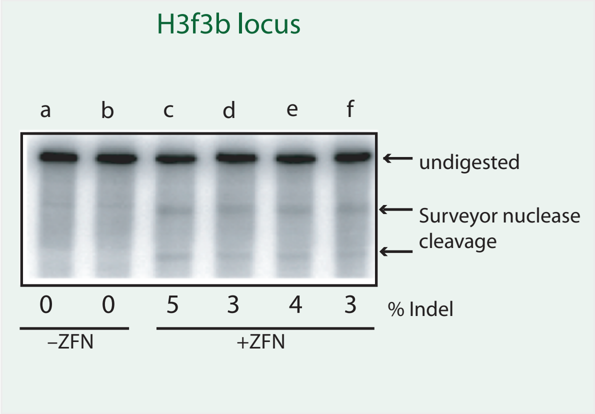

Supplement: Figure S3 — Analysis of imprecise intrachromosomal NHEJ at the ZFNRosa26 and ZFNH3f3b loci. A) Surveyor nuclease assay for Lig3 null and nuclear Lig3-deficient cells. Genomic DNA from ES cells transfected with no ZFN or both ZFNRosa26 and ZFNH3f3b that was used to quantify translocation frequency is also used as a template to amplify across the cleavage site at either the Rosa26 or H3f3b locus to quantify intrachromosomal NHEJ. After PCR, the amplification products are denatured and then reannealed to form heteroduplexes between unmodified and modified sequences from imprecise NHEJ. The mismatched duplex is selectively cleaved by Surveyor nuclease at the loops that form at the site of the mismatch. The percentage of locus modification from insertion/deletion (% Indel) is indicated at the bottom of each sample and provides an estimate imprecise of NHEJ at the Rosa26 and H3f3b loci. Given that the assay is sensitive to 1% locus modification, similar levels of imprecise NHEJ are observed for all cell lines tested. B) Analysis of intrachromosomal NHEJ by bacterial colony hybridization. The amplified product from (A) is also cloned using the TOPO-TA cloning system and transformed into bacteria. Bacterial colonies are hybridized with probes for unmodified Rosa26 or H3f3b ZFN target sequences. Colonies that hybridize with either of these probes will be from unmodified loci, while those that do not are from modified loci arising from imprecise NHEJ. Therefore, percent imprecise NHEJ (% Indel) at each locus is calculated as ratio of the number of colonies that do not hybridize with either of these probes to the total number of colonies analyzed. Plasmids from colonies that do not hybridize are sequenced to confirm that they contain imprecise NHEJ events. The imprecise NHEJ frequency obtained with this method is similar to the Surveyor nuclease assay results. C) Surveyor nuclease assay for the Lig1 depletion experiments. Similar levels of imprecise NHEJ were observed in the nuclear Lig3- [file pgen.1002080.s003.pdf]
