# Supplementary material for: DNA Ligase III Promotes Alternative Nonhomologous End-Joining during Chromosomal Translocation Formation
Source: PLoS Genet. 2011 Jun 2;7(6):e1002080. doi: 10.1371/journal.pgen.1002080 (PMC3107202; doi:10.1371/journal.pgen.1002080)
Supplement: Figure S4 — Statistics for microhomology distribution. A two-tailed Mann-Whitney test was applied, with P values derived from a comparison with Lig3KO/KO; Lig3 GFP (a) and Expected by chance (b). (PDF) [file pgen.1002080.s004.pdf]

Figure S4

statistics for microhomology distribution	*P value* a	b
$Lig3^{KO/KO}$;		
Lig3 GFP		<0.0001
Lig3 GFP + Lig1 shRNA	0.7859	<0.0001
$Xrcc4^{-/-}$	0.6905	<0.0001
$Lig3^{KO/KO}$;		
MtLig3 ΔBRCT GFP NES	<0.0001	0.6935
MtLig1 GFP	0.0008	0.0834
MtLig1 ΔNLS GFP	0.0002	0.2616
Lig3 ΔBRCT GFP	0.9112	<0.0001
Lig3 ΔZNF GFP	0.3426	<0.0001
