# Supplementary material for: DNA Ligase III Promotes Alternative Nonhomologous End-Joining during Chromosomal Translocation Formation
Source: PLoS Genet. 2011 Jun 2;7(6):e1002080. doi: 10.1371/journal.pgen.1002080 (PMC3107202; doi:10.1371/journal.pgen.1002080)
Supplement: Figure S5 — Der(6) translocation junction sequences obtained from the indicated genotypes. ZFN recognition sites are indicated in red (chr. 6) and blue (chr. 11), with fill-in of the 5′ overhang for chr. 6. The cleavage site for chr. 11 is likely to be variable in whether it includes the terminal G (in parentheses) or not; therefore, the terminal G was not scored as an insertion in the junctions. Sequences are annotated as follows: del, deletion length from the DNA end after DSB formation; underline, microhomology; middle black letters, sequence of short insertion; +, length of long insertion. If the deletion extends beyond the chromosome sequence indicated at the top of the figure, a few bp flanking the deletion are indicated, with microhomology underlined. (PDF) [file pgen.1002080.s005.pdf]

Figure S5

A) *Lig3*$^{KO/cKOneo+}$

Translocation clones	Chr. 6	Translocation junctions	Chr. 11
	CAGAAGACTCCCGCCCATCTTC		(G)CTCGCCGGATACGGGGGAGAGAGC
E2bE1	CAGAAGACTCCCGCCCATCTTC		GCTCGCCGGATACGGGGGAGAGAGC
E2bG2	CAGAAGACTCCCGCCCATCTT		CTCGCCGGATACGGGGGAGAGAGC
E1bH3	CAGAAGACTCCCGCCCATCT		GCTCGCCGGATACGGGGGAGAGAGC
E1E11	CAGAAGACTCCCGCCCATCT	+185 inser.	GCTCGCCGGATACGGGGGAGAGAGC
E2bA1	CAGAAGACTCCCGCCCATC		CTCGCCGGATACGGGGGAGAGAGC
E1C8	CAGAAGACTCCCGCCCATCTTC		GCCGGATACGGGGGAGAGAGC
E2D1	CAGAAGACTCCCGCCCATCTTC		GCCGGATACGGGGGAGAGAGC
E2bD5	CAGAAGACTCCCGCCCATCTTC		GCCGGATACGGGGGAGAGAGC
E2bE2	CAGAAGACTCCCGCCCATCTTC	TCGATACGGA	CGGATACGGGGGAGAGAGC
E1H8	CAGAAGACTCCCGCCCAT		GCTCGCCGGATACGGGGGAGAGAGC
E2B6	CAGAAGACTCCCGCCCAT		GCTCGCCGGATACGGGGGAGAGAGC
E2H12	CAGAAGACTCCCGCCCAT		GCTCGCCGGATACGGGGGAGAGAGC
E1G6	CAGAAGACTCCCGCCCATCT		CGCCGGATACGGGGGAGAGAGC
E2C2	CAGAAGACTCCCGCCCATCT		CGCCGGATACGGGGGAGAGAGC
E2A12	CAGAAGACTCCCGCCCATCT		CGCCGGATACGGGGGAGAGAGC
E2bB1	CAGAAGACTCCCGCCCATCT		CGCCGGATACGGGGGAGAGAGC
E2F9	CAGAAGACTCCCGCCCATCT		GCCGGATACGGGGGAGAGAGC
E2A1	CAGAAGACTCCCGCCC		GCTCGCCGGATACGGGGGAGAGAGC
E2bF6	CAGAAGACTCCCGCCC		GCTCGCCGGATACGGGGGAGAGAGC
E2E11	CAGAAGACTCCCGCCCATC	GTATC	CGGATACGGGGGAGAGAGC
E1E9	CAGAAGACTCCCGCCCATCT		CGGATACGGGGGAGAGAGC
E2bC3	CAGAAGACTCCCGCCC		TCGCCGGATACGGGGGAGAGAGC
E2bC8	CAGAAGACTCCCGCCC		TCGCCGGATACGGGGGAGAGAGC
E1F7	CAGAAGACTCCCGCCCATC		CGGATACGGGGGAGAGAGC
E1B7	CAGAAGACTCCCGCCCAT	GCTCA	CCGGATACGGGGGAGAGAGC
E1bE2	CAGAAGACTCCCGCCCA	AAA	CCGGATACGGGGGAGAGAGC
E2bE12	CAGAAGACTCCCGCCCA	AAAAAAAA	CCGGATACGGGGGAGAGAGC
E2H7	CAGAAGACTCCCGCCC		CGGATACGGGGGAGAGAGC
E2F1	CAGAAGACTCCCGCC	T	CCGGATACGGGGGAGAGAGC
E1G11	CAGAAGACTCCCGCCC		GGATACGGGGGAGAGAGC
E2F7	CAGAAGACTCCCGCCC		GGATACGGGGGAGAGAGC
E2bG7	CAGAAGACTCCCGCCC		GGATACGGGGGAGAGAGC
E2bE10	CAGAAGACTCCCGCC	+12 inser.	CGGATACGGGGGAGAGAGC
E1H4	CAGAAGACTCCCGCCC	+20 inser.	GGATACGGGGGAGAGAGC
E1F3	CAGAAGACTCCCGCC		GGATACGGGGGAGAGAGC
E1B8	CAGAAGACTCCCGCC		GGATACGGGGGAGAGAGC
E1D9	CAGAAGACTCCCGCC		GGATACGGGGGAGAGAGC
E2G10	CAGAAGACTCCCGCC		GGATACGGGGGAGAGAGC
E2bD4	CAGAAGACTCCCGCC		GGATACGGGGGAGAGAGC
E2bE7	CAGAAGACTCCCGCC		GGATACGGGGGAGAGAGC
E1G1	CAGAAGACTCCCGC		GGATACGGGGGAGAGAGC
E2E2	CAGAAGACTCCCG		GGGGAGAGAGC
E2bG12	CAGAAGACTC		GCCGGATACGGGGGAGAGAGC
E1C10	CAGAAGACTCCCGCCCATC		GGGAGAGAGC
E2bF9	CAGAAG		GCTCGCCGGATACGGGGGAGAGAGC
E1bF7	CAGAAGACTCCCGCCCA		CGGGGGAGAGAGC
E2H9	CAGAAGACTCCCG		GATACGGGGGAGAGAGC
E2bH5	CAGAAGACTCCCG		GATACGGGGGAGAGAGC
E2bC10	CAGAAGACTCCCG		GATACGGGGGAGAGAGC
E1F2	CAGAAGACTCC		GGATACGGGGGAGAGAGC
E1G3	CAGAAGACTCCCGC		GGGGGAGAGAGC
E2E12	CAGAAGACTCCCGCCCA		GGGAGAGAGC
E1B12	CAGAAGACTCCCG		GGGGGAGAGAGC

Figure S5, con't.

A) *Lig3*$^{KO/cKOneo+}$

	Chr. 6		Chr. 11	
	CAGAAGACTCCCGCCCATCTTC		(G)CTCGCCGGATACGGGGGAGAGAGC	
Translocation clones		Translocation junctions		
E1H3	CAGAAGACTCCCG		GATACGGGGGAGAGAGC	
E1A5	CAGAAGACTCCCG		GATACGGGGGAGAGAGC	
E1bB3	CAGAAGACTCCCGCCCAT		GAGC	
E1B6	CAGAAGAC		CGGATACGGGGGAGAGAGC	
E2C5	CAGAAGACTCCCGCC	TTA	AGAGAGC	
E2bA5	CAGAAGAC		GGGGGAGAGAGC	
E2bH5	CCC		GATACGGGGGAGAGAGC	
E1bA7	CAGAAGACT	G	CGGGGGAGAGAGC	
E1C9	CAGA		GAGC	
E1B10	GGT -39del		GCTCGCCGGATACGGGGGAGAGAGC	
E1G12	CAGAAGACTCCCGC		-38del	GGT
E2C1	AAG -27del		AGAGC	
E2F4	AGC -26del	+36 inser.		AGC
E2bC6	CAGAAG		-37del	CGG
E1bD12	CAGAAG		-37del	CGG
E1H2	TTTAAG		-30del	TTG
E1D5	TCC -68del	+48 inser.	GATACGGGGGAGAGAGC	
E1H5	ACC -78del		GGATACGGGGGAGAGAGC	
E2bC11	ACA -47del		-52del	TTT
E1G5	AAA -135del	+432 inser.	CCGGATACGGGGGAGAGAGC	
E2bC6	CAGAAGACTCCCG		GATACGGGGGAGAGAGC	
E1E2	CAGAAGACTCCCGCCCATCTT	+67 inser.	-84del	TTA

Figure S5, con't.

B) *Lig3$^{KO/KO}$; MtLig3 ΔBRCT GFP NES*

Translocation clones	Chr. 6	Translocation junctions	Chr. 11
	CAGAAGACTCCCGCCCATCTTC		(G)CTCGCCGGATACGGGGGAGAGAGC
E1E10	CAGAAGACTCCCGCCCATCTTC		CTCGCCGGATACGGGGGAGAGAGC
E3D1	CAGAAGACTCCCGCCCATCTTC		GCCGGATACGGGGGAGAGAGC
E1C9	CAGAAGACTCCCGCCCATCTTC		CCGGATACGGGGGAGAGAGC
E3G9	CAGAAGACTCCCGCCCAT		GCTCGCCGGATACGGGGGAGAGAGC
E4F2	CAGAAGACTCCCGCCCAT	GA	GCTCGCCGGATACGGGGGAGAGAGC
E1E2	CAGAAGACTCCCGCCCATCT		GCCGGATACGGGGGAGAGAGC
E1D4	CAGAAGACTCCCGCCCATC		GCCGGATACGGGGGAGAGAGC
E1H9	CAGAAGACTCCCGCCCATC		GCCGGATACGGGGGAGAGAGC
E1H5	CAGAAGACTCCCGCCCA		CGCCGGATACGGGGGAGAGAGC
E1E11	CAGAAGACTCCCGCCCAT		CCGGATACGGGGGGAGAGAGC
E3F1	CAGAAGACTCCCGCCCA		CCGGATACGGGGGAGAGAGC
E1G1	CAGAAGACTCCCGCCC		GCCGGATACGGGGGAGAGAGC
E2E12	CAGAAGACTCCC	+225 bp	CTCGCCGGATACGGGGGAGAGAGC
E1F3	CAGAAGACTCC	GC	CCGGATACGGGGGAGAGAGC
E2B5	CAGAAGACTCCCGCCCATCTTC	G	GGGGGAGAGAGC
E3G3	CAGAAGACTCCCGCCCA		TACGGGGGAGAGAGC
E2F8	CAGAAGAC		CGGATACGGGGGAGAGAGC
E3F8	CAGAAGACT	T	CCGGATACGGGGGAGAGAGC
E4H1	CAGAAGACTCC		GATACGGGGGAGAGAGC
E2G10	CAGAAGACTCCCGCCC		GGGGGAGAGAGC
E3H3	CAGAAGACTCC	A	GATACGGGGGAGAGAGC
E1A8	CAGAAGA		GGATACGGGGGAGAGAGC
E1F2	CAGAAGACTCCCGCCC		GGGGGAGAGAGC
E3A6	CAG		GCCGGATACGGGGGAGAGAGC
E3F3	CAGAAGACT		TACGGGGGAGAGAGC
E4H2	CAGAAGACTCCCG		GGGGAGAGAGC
E4D8	CAGAAGACTCCCGCCCA	AGCTACGG	AGAGAGC
E4G8	CAGA		TACGGGGGAGAGAGC
E4E9	CAGAAGACTCCCG		GAGAGC
E4H5	CAGA		CGGGGGAGAGAGC
E3F2	CAGAAGACT		GC
E1C1	CAGGT -40 del		ACGGGGGAGAGAGC
E3C5	CAGAAGACTC		-69 del TAAG[illegible]
E2H8	TTCAA -73 del	+69 bp	ACGGGGGAGAGAGC
E3D9	GTTA -37 del		-56 del AGTA
E2C3	GCCTG -24 del		-82 del AATT[illegible]
E4A6	GAAGT -101 del		-64 del GCAAG

Figure S5, con't.

C) *Lig3*$^{KO/KO}$*; MtLig1 GFP*

Translocation clones	Chr. 6	Translocation junctions	Chr. 11	
	CAGAAGACTCCCGCCCATCTTC		(G)CTCGCGGATACGGGGGAGAGAGC	
E2E1	CAGAAGACTCCCGCCCATCTTC	TCT	TCGCCGGATACGGGGGAGAGAGC	
E1C12	CAGAAGACTCCCGCCCATCTTC		GCTCGCCGGATACGGGGGAGAGAGC	
E1bG10	CAGAAGACTCCCGCCCATCTTC		CGCCGGATACGGGGGAGAGAGC	
E1F1	CAGAAGACTCCCGCCCATCTTC		GCCGGATACGGGGGAGAGAGC	
E1D4	CAGAAGACTCCCGCCCATCTTC		GCCGGATACGGGGGAGAGAGC	
E1A10	CAGAAGACTCCCGCCCAT		GCTCGCCGGATACGGGGGAGAGAGC	
E2G8	CAGAAGACTCCCGCCCAT		GCTCGCCGGATACGGGGGAGAGAGC	
E1bE5	CAGAAGACTCCCGCCCATCTTC		CCGGATACGGGGGAGAGAGC	
E2A10	CAGAAGACTCCCGCCCATCT		CGCCGGATACGGGGGAGAGAGC	
E1E7	CAGAAGACTCCCGCCCATCT		CGCCGGATACGGGGGAGAGAGC	
E2bF9	CAGAAGACTCCCGCCCATCT		CGCCGGATACGGGGGAGAGAGC	
E2bC4	CAGAAGACTCCCGCCCATCT		GCCGGATACGGGGGAGAGAGC	
E2bE12	CAGAAGACTCCCGCC		TCGCCGGATACGGGGGAGAGAGC	
E2H10	CAGAAGACTCCCGC		TCGCCGGATACGGGGGAGAGAGC	
E2H5	CAGAAGACTCCCGCCCA	AGACAT	CCGGATACGGGGGAGAGAGC	
E1F4	CAGAAGACTCCCGCCC		CCGGATACGGGGGAGAGAGC	
E2bD2	CAGAAGACTCCCGCCCAT		ATACGGGGGAGAGAGC	
E1A3	CAGAAGACTCCCGCCCATCT		ACGGGGGAGAGAGC	
E1D9	CAGAAGACTCCCGCCC		GGATACGGGGGAGAGAGC	
E2bH3	CAGAAGACTCCCGCCC		GGATACGGGGGAGAGAGC	
E1A1	CAGAAGACTCCCGCCCATC		GGGGGAGAGAGC	
E1H12	CAGAAGACTCCCGCCCATC		ACGGGGGAGAGAGC	
E1A6	CAGAAGACTCCCGCC		GGATACGGGGGAGAGAGC	
E2C4	CAGAAGACTCCCGCCCATCTTCT		GGGAGAGAGC	
E1F9	CAGAAGACTCCCGCC		GATACGGGGGAGAGAGC	
E2bA12	CAGAAGACTCCCGCCCA		CGGGGGAGAGAGC	
E1C9	CAGAAGACTCCCG		GATACGGGGGAGAGAGC	
E1C10	CAGAAGACTCCCG		GATACGGGGGAGAGAGC	
E2B8	CAGAAGACTCCCGC		GGGGAGAGAGC	
E1E11	CAGAAGACTCCCG		ATACGGGGGAGAGAGC	
E1G10	CAGAAGACTCCCGCCCA		GGGGAGAGAGC	
E1H8	CAGAAGACTCCCGCCC		GGGGGAGAGAGC	
E1D3	CAGAAGACTCCCGCCCATCTTC	GC	GAGAGC	
E2bB12	CAGAAGAC		GGATACGGGGGAGAGAGC	
E1C11	CAGAAGACTCCCGCCCATCTTCT	G	GAGC	
E1F2	CAGAAGA		TACGGGGGAGAGAGC	
E1H1	CCC		GGATACGGGGGAGAGAGC	
E1E3	CAGAAGAC		GGGGAGAGAGC	
E1bF2	CAGA		TACGGGGGAGAGAGC	
E1G4	CAGAAGACTCCCG		AGAGC	
E2G6	CAGAAGACTCCCGCCC		-26del	TTA
E1G2	CAGAAGA		GAGAGAGC	
E1G5	CAGAAGACTC	AAG	AGAGC	
E2E11	CTG -26del	GAACTTC	ATACGGGGGAGAGAGC	
E2bF11	CCT -27del	+160 inser.	CGGGGGAGAGAGC	
E2bC1	CAGAAG		-26del	TTA
E2G7	CAGA			CTT
E1H6	CAGAAGACTCCCGCCCAT		-81del	AAA
E1H11	TTA -33del	+29 inser.	-56del	TGT
E1bE6	TCT -111del	+36 inser.	GCCGGATACGGGGGAGAGAGC	

Figure S5, con't.

D) *Lig3$^{KO/KO}$; MtLig1 ΔNLS GFP*

Translocation clones	Chr. 6<br>CAGAAGACTCCCGCCCATCTTC	Translocation junctions	Chr. 11<br>(G)CTCGCCGGATACGGGGGAGAGAGC
E1E1	CAGAAGACTCCCGCCCATCTTC		GCTCGCCGGATACGGGGGAGAGAGC
E1H12	CAGAAGACTCCCGCCCATCTT		TCGCCGGATACGGGGGAGAGAGC
E2E9	CAGAAGACTCCCGCCCATCT		CGCCGGATACGGGGGAGAGAGC
E1B2	CAGAAGACTCCCGCCCATCT		TCGCCGGATACGGGGGAGAGAGC
E2A2	CAGAAGACTCCCGCCCATCT		TCGCCGGATACGGGGGAGAGAGC
E1F9	CAGAAGACTCCCGCCCAT	T	GCTCGCCGGATACGGGGGAGAGAGC
E1G5	CAGAAGACTCCCGCCCATCT		CGCCGGATACGGGGGAGAGAGC
E2C10	CAGAAGACTCCCGCCCAT		GCTCGCCGGATACGGGGGAGAGAGC
E2A10	CAGAAGACTCCCGCCCA		CGCCGGATACGGGGGAGAGAGC
E2B8	CAGAAGACTCCCGCCCATC		GCCGGATACGGGGGAGAGAGC
E2G8	CAGAAGACTCCCGCCCATCT		GGATACGGGGGAGAGAGC
E2B10	CAGAAGACTCCCGCCCATC		CGGATACGGGGGAGAGAGC
E2B8	CAGAAGACTCCCGCCCATC		CGGATACGGGGGAGAGAGC
E2C1	CAGAAGACTCCCGCCCATC		CGGATACGGGGGAGAGAGC
E2B12	CAGAAGACTCCCGCCC		GCCGGATACGGGGGAGAGAGC
E1D12	CAGAAGACTCCCGCCCA		GATACGGGGGAGAGAGC
E1G11	CAGAAGACTCCCGCCCATC		GGATACGGGGGAGAGAGC
E2A4	CAGAAGACTCCCGCCCATC		GGATACGGGGGAGAGAGC
E1G9	CAGAAGACTCCCGCCC		GGATACGGGGGAGAGAGC
E2D6	CAGAAGACTCCCGCC		GGATACGGGGGAGAGAGC
E2D1	CAGAAGACT	TTC	GCTCGCCGGATACGGGGGAGAGAGC
E2H6	CAGAAGACTCCCG	TAT	CCGGATACGGGGGAGAGAGC
E3C2	CAGAAGACTCCCGCC		GGATACGGGGGAGAGAGC
E2B7	CAGAAGACTCCCGCCCA		CGGGGGAGAGAGC
E1H4	CAGAAGACTCCCGC		ATACGGGGGAGAGAGC
E2B5	CAGAAG		CTCGCCGGATACGGGGGAGAGAGC
E3C7	CAGAAGACTCCCG		GATACGGGGGAGAGAGC
E1D5	CAGAAGACTCCCGCCC	GT	ACGGGGGAGAGAGC
E2B11	CAGAAGACTC		GGGGGAGAGAGC
E1A5	CAGAA		CCGGATACGGGGGAGAGAGC
E1C8	CAGAAGACTCCCGCCC		GGGAGAGAGC
E1D7	CAGAAGACTCCCGCCCATC	CAGTTCAGACCT	AGAGAGC
E2D12	CAGAAGACTCCC		GGGGGAGAGAGC
E2C3	CAGAAGACT		ACGGGGGAGAGAGC
E3B5	CAGAAGACTCCCGCCCA		AGC
E2H3	CAGAAGAC		GGGGGAGAGAGC
E2E5	CAG		GAGAGAGC
E1B11	CAGAAGACTCCCG	TATA	C
E2A8	CAGAAGACTCCCGC	AT	-35 del GCG[illegible]
E1A8	C		GC
E1A11	CTGCC -22 del	GGGGGGAGGCAGGGC	AGC
E3D3	GCAGG -60 del		ATACGGGGGAGAGAGC
E2B1	CACCA -42 del		-44 del TATGG
E1C7	CAGAAGACTCCCG		-89 del GTTT[illegible]
E2G9	TGGAA -92 del		CGGGGGAGAGAGC
E2H10	CGTTA -37 del		-69 del AGTAA
E1B1	CAGAAGACTC	+174 bp	-163 del AATG[illegible]

Figure S5, con't.

E) *Lig3^{KO/KO}*; *Lig3 GFP*

Translocation clones	Chr. 6<br>CAGAAGACTCCCGCCCATCTTC	Translocation junctions	Chr. 11<br>(G)CTCGCCGGATACGGGGGAGAGAGC
E2D10	CAGAAGACTCCCGCCCATCTTC		GCTCGCCGGATACGGGGGAGAGAGC
E3F3	CAGAAGACTCCCGCCCATCTTC		GCTCGCCGGATACGGGGGAGAGAGC
E4F5	CAGAAGACTCCCGCCCATCTTC		CTCGCCGGATACGGGGGAGAGAGC
E3D11	CAGAAGACTCCCGCCCATCTTC		TCGCCGGATACGGGGGAGAGAGC
E4B2	CAGAAGACTCCCGCCCATCTTC		TCGCCGGATACGGGGGAGAGAGC
E4G6	CAGAAGACTCCCGCCCATCT		CTCGCCGGATACGGGGGAGAGAGC
E5E4	CAGAAGACTCCCGCCCATCT	CC	CTCGCCGGATACGGGGGAGAGAGC
E2C9	CAGAAGACTCCCGCCCATCTTC		GCCGGATACGGGGGAGAGAGC
E4B4	CAGAAGACTCCCGCCCATCTTC		GCCGGATACGGGGGAGAGAGC
E5G11	CAGAAGACTCCCGCCCATCTTC		GCCGGATACGGGGGAGAGAGC
E4G12	CAGAAGACTCCCGCCCATCT		CGCCGGATACGGGGGAGAGAGC
E5G12	CAGAAGACTCCCGCCCAT		TCGCCGGATACGGGGGAGAGAGC
E4H3	CAGAAGACTCCCGCCCAT		CGCCGGATACGGGGGAGAGAGC
E4F2	CAGAAGACTCCCGCCCATC	G	GCCGGATACGGGGGAGAGAGC
E3C2	CAGAAGACTCCCGCCC		TCGCCGGATACGGGGGAGAGAGC
E3H7	CAGAAGACTCCCGCCC		TCGCCGGATACGGGGGAGAGAGC
E5C10	CAGAAGACTCCCGCCCA	AAGACAT	CCGGATACGGGGGAGAGAGC
E4E8	CAGAAGACTCCCGCCCA	A	CCGGATACGGGGGAGAGAGC
E2F12	CAGAAGACTCCCGCCCA		CGGATACGGGGGAGAGAGC
E2E8	CAGAAGACTCCCGCCC	+21bp	CGGATACGGGGGAGAGAGC
E2G8	CAGAAGACTCCCGCCCATCT	CGGCCGGG	TACGGGGGAGAGAGC
E4A5	CAGAAGACTCCCGCCC	TGTCAGTTAA	CGGATACGGGGGAGAGAGC
E5B11	CAGAAGACTC		TCGCCGGATACGGGGGAGAGAGC
E2E1	CAGAAGACTCCCGCC		GGATACGGGGGAGAGAGC
E3F2	CAGAAGACTCCCGCC		GGATACGGGGGAGAGAGC
E3F4	CAGAAGACTCCCGCC		GGATACGGGGGAGAGAGC
E3G11	CAGAAGACTCCCGCC		GGATACGGGGGAGAGAGC
E3B12	CAGAAGACTCCCGCC		GGATACGGGGGAGAGAGC
E4B8	CAGAAGACTCCCGCC		GGATACGGGGGAGAGAGC
E5H2	CAGAAGACTCCCGCC		GGATACGGGGGAGAGAGC
E5B7	CAGAAGACTCCCGCC		GGATACGGGGGAGAGAGC
E4B10	CAGAAGACTCCCGCCCATCT		GGGGGAGAGAGC
E2F6	CAGAAGACTCCCG		GGGGAGAGAGC
E3C5	CAGAAGACTCCC	T	CCGGATACGGGGGAGAGAGC
E2C6	CAGAAGACTCCCGCC		ATACGGGGGAGAGAGC
E4E11	CAGAAGACTCC		CGGATACGGGGGAGAGAGC
E5A9	CAGAAGACTCCCGC	A	GATACGGGGGAGAGAGC
E4E7	CAGAAGACTCCC		GGATACGGGGGAGAGAGC
E2E11	CAGAAGACTCCCG		GATACGGGGGAGAGAGC
E5F9	CAGAAGACTCCCG		GATACGGGGGAGAGAGC
E2A7	CAGAAGACTCCCG		GATACGGGGGAGAGAGC
E4D2	CAGAAGACTCCCGCCC	CGG	GGGGGAGAGAGC
E3F5	CAGAAGACT		GGATACGGGGGAGAGAGC
E3A12	CAGAAG		CCGGATACGGGGGAGAGAGC
E3H12	CAGAAGA	TAC	GGATACGGGGGAGAGAGC
E5E9	CAGAA	AACTC	CCGGATACGGGGGAGAGAGC
E2D9	CAGAAGACTCCC	TCGGATCA	GGGGAGAGAGC
E5D3	CAGAAGACTCCCG		GGGAGAGAGC

Figure S5, con't.

E) *Lig3*$^{KO/KO}$; *Lig3 GFP*

Translocation clones	Chr. 6<br>CAGAAGACTCCCGCCCATCTTC	Translocation junctions	Chr. 11<br>(G)CTCGCCGGATACGGGGGGAGAGAGC
E2B2	CAGAAGA		TACGGGGGGAGAGAGC
E4F7	CAGAAGA		TACGGGGGGAGAGAGC
E4C12	CAGAAGA		TACGGGGGGAGAGAGC
E2C10	CAGAAG		TACGGGGGGAGAGAGC
E2A12	CAGAAGACT	G	CGGGGGGAGAGAGC
E3D6	CAG		GATACGGGGGGAGAGAGC
E5A12	CAGAAGAC		GGGGGGAGAGAGC
E5E11	CAGAAGACTCCCGCCCAT		-25 del TTAAG
E5G8	CAGA		TACGGGGGGAGAGAGC
E3H5	TGCC -22del		GGATACGGGGGGAGAGAGC
E2C7	CAGAAGA	A	GGGGGAGAGAGC
E3D1	CAGAAGACTCCCGCCC	+265bp	AGC
E5B2	CAGAAGA	TACA	GGGGGAGAGAGC
E4C3	CAGAAGACTCCCGCCCAT	G	-28 del AGTTC
E4G11	CCTG -24del		GGGGGAGAGAGC
E4A4	CAGAAGACT		-26 del TAAGT
E3B10	CAGAA		-27 del AAGTT
E5B4	CAGAAG		-30 del TTGAA
E3H9	CAGAAG		-37 del CGGTT
E2H1	TTAAG -29 del		-30 del TTGAA
E4H1	TAGC -35 del		-25 del TTAAG
E4H4	TAGC -35 del		-25 del TTAAG
E3G1	GTTA -37 del		-28 del AGTTC
E2G10	CCGG -68 del		GGGGAGAGAGC
E4F4	CAGAAGACTCCCG		-140 del TCATT
E5A3	ACCT -78 del		-84 del TTGGT

Figure S5, con't.

F) *Lig3*$^{KO/KO}$; *Lig3 ΔZnF GFP*

Translocation clones	Chr. 6	Translocation junctions	Chr. 11
	CAGAAGACTCCCGCCCATCTTC		(G)CTCGCCGGATACGGGGGAGAGAGC
E4D6	CAGAAGACTCCCGCCCATCTTC		TCGCCGGATACGGGGGAGAGAGC
E4H4	CAGAAGACTCCCGCCCATCTTC		GCCGGATACGGGGGAGAGAGC
E7D11	CAGAAGACTCCCGCCCATCTTC		GCCGGATACGGGGGAGAGAGC
E5H2	CAGAAGACTCCCGCCCATCTTC	+30 bp	GCCGGATACGGGGGAGAGAGC
E4C5	CAGAAGACTCCCGCCC		TCGCCGGATACGGGGGAGAGAGC
E1A5	CAGAAGACTCCCGCCCATCT		CGCCGGATACGGGGGAGAGAGC
E2G2	CAGAAGACTCCCGCCCATCT		CGCCGGATACGGGGGAGAGAGC
E5E5	CAGAAGACTCCCGCCCATCT		CGCCGGATACGGGGGAGAGAGC
E1C3	CAGAAGACTCCCGCCCAT	G	GCTCGCCGGATACGGGGGAGAGAGC
E5A3	CAGAAGACTCCCGCCCATC		GCCGGATACGGGGGAGAGAGC
E2G12	CAGAAGACTCCCGCCCATC	CAGTT	GCCGGATACGGGGGAGAGAGC
E5C4	CAGAAGACTCCCGCCCAT		GCCGGATACGGGGGAGAGAGC
E4B1	CAGAAGACTCCCGCCCA	+288 bp	CGCCGGATACGGGGGAGAGAGC
E3A6	CAGAAGACTCCCGCCCATC		CGGATACGGGGGAGAGAGC
E1E6	CAGAAGACTCCCGCCCATCT	+25 bp	GGATACGGGGGAGAGAGC
E5F12	CAGAAGACTCCCGCCC	+300 bp	GCCGGATACGGGGGAGAGAGC
E7A6	CAGAAGACTCCCGCCC		CCGGATACGGGGGAGAGAGC
E2D7	CAGAAGACTCCCGCCC		CGGATACGGGGGAGAGAGC
E6C10	CAGAAGACTCCC		TCGCCGGATACGGGGGAGAGAGC
E4G7	CAGAAGACTCCCG		CCGGATACGGGGGAGAGAGC
E1D8	CAGAAGACTCCCGCC		GGATACGGGGGAGAGAGC
E1A9	CAGAAGACTCCCGCC		GGATACGGGGGAGAGAGC
E2A4	CAGAAGACTCCCGCC		GGATACGGGGGAGAGAGC
E3B9	CAGAAGACTCCCGCC		GGATACGGGGGAGAGAGC
E5D2	CAGAAGACTCCCGCC		GGATACGGGGGAGAGAGC
E6A1	CAGAAGACTCCCGCC		GGATACGGGGGAGAGAGC
E7C4	CAGAAGACTCCCGCC		GGATACGGGGGAGAGAGC
E2E2	CAGAAGACTCCCGCCCAT	TG	GGGGGAGAGAGC
E7C10	CAGAAGACTCCCGCCCATCTTC		GGGGGAGAGAGC
E6B11	CAGAAGACTCCCGC	+312 bp	GGATACGGGGGAGAGAGC
E6A3	CAGAAGACTC		GCCGGATACGGGGGAGAGAGC
E2B9	CAGAAGACTCCCG		GATACGGGGGAGAGAGC
E5F10	CAGAAGACTCCCG		GATACGGGGGAGAGAGC
E6A4	CAGAAGACTCCCG		GATACGGGGGAGAGAGC
E7A4	CAGAAGACTCCCG		GATACGGGGGAGAGAGC
E6A11	CAGAAGACTCC		GGATACGGGGGAGAGAGC
E6D1	CAGAAGACT	+261 bp	CCGGATACGGGGGAGAGAGC
E7F7	CAGAAGACTCCCGCCCAT		GGGGAGAGAGC
E3B5	CAGAAG	+260 bp	CCGGATACGGGGGAGAGAGC
E4C8	CAGAAGACTCCCG		GGGGGAGAGAGC
E3D9	CAGAAGACTCCCG		GGGGAGAGAGC
E2C11	CAGAAG		C
E4G9	CAGAAGACTCCC		ATACGGGGGAGAGAGC
E3D7	CAGAAGACTCCCGCCCA		GAGC
E2G7	CAGAAGACTC	+23 bp	GAGAGAGC
E7F5	CAGAAGA		GAGC

Figure S5, con't.

F) *Lig3*$^{KO/KO}$; *Lig3 ΔZnF GFP*

Translocation clones	Chr. 6<br>CAGAAGACTCCCGCCCATCTTC	Translocation junctions	Chr. 11<br>(G)CTCGCCGGATACGGGGGGAGAGAGC
E6E1	CAGA		GGAGAGAGC
E3H10	CAGAAGA		GAGC
E4F12	CAGA		GAGAGC
E7D4	AGCCT -25 del	TTTTCA	GAGAGAGC
E2H11	CAGAAG		-37 del CGGGT
E7B12	CAGAAG		-37 del CGGTT
E2H12	CAGAAGACTCCCGCCCA	+75 bp	-66 del GTAAA
E6D12	TAGC -35 del		-38 del GGTTT
E7B2	CCAGG -39 del		-47 del CATTT
E1H7	TCCAA -88 del		GATACGGGGGGAGAGAGC
E1B8	ACCG -118 del		GATACGGGGGGAGAGAGC
E3C3	CAGAAGACTCCCGC	+152 bp	-132 del ACTTA
E7B8	CCCAT -158 del	+142 bp	GGGGAGAGAGC
E3B12	CAG		-199 del TGATG

Figure S5, con't.

G) *Lig3*$^{KO/KO}$; *Lig3 ΔBRCT GFP*

Translocation clones	Chr. 6	Translocation junctions	Chr. 11
	CAGAAGACTCCCGCCCATCTTC		(G)CTCGCCGGATACGGGGGAGAGAGC
E3C5	CAGAAGACTCCCGCCCATCTTC		CTCGCCGGATACGGGGGAGAGAGC
E1E3	CAGAAGACTCCCGCCCATCTTC		TCGCCGGATACGGGGGAGAGAGC
E2B7	CAGAAGACTCCCGCCCATCTTC		TCGCCGGATACGGGGGAGAGAGC
E2G10	CAGAAGACTCCCGCCCATCTTC		GCCGGATACGGGGGAGAGAGC
E3A2	CAGAAGACTCCCGCCCATCTTC		GCCGGATACGGGGGAGAGAGC
E1A1	CAGAAGACTCCCGCCCATC		GCTCGCCGGATACGGGGGAGAGAGC
E3C4	CAGAAGACTCCCGCCCATC	GC	GCTCGCCGGATACGGGGGAGAGAGC
E1E2	CAGAAGACTCCCGCCCATCT		CGCCGGATACGGGGGAGAGAGC
E2D4	CAGAAGACTCCCGCCCAT	A	GCTCGCCGGATACGGGGGAGAGAGC
E3D1	CAGAAGACTCCCGCCCAT		GCTCGCCGGATACGGGGGAGAGAGC
E1C2	CAGAAGACTCCCGCCCATCT		GCCGGATACGGGGGAGAGAGC
E3F1	CAGAAGACTCCCGCCCATCTTC		CGGATACGGGGGAGAGAGC
E2F8	CAGAAGACTCCCGCCCATC		GCCGGATACGGGGGAGAGAGC
E3C3	CAGAAGACTCCCGCCCATC		GCCGGATACGGGGGAGAGAGC
E1D1	CAGAAGACTCCCGCCC	+28 bp	CGCCGGATACGGGGGAGAGAGC
E3B6	CAGAAGACTCCCGCCCATC		GGATACGGGGGAGAGAGC
E3F6	CAGAAGACTCCCGCCCA		CGGATACGGGGGAGAGAGC
E1F6	CAGAAGACTCCCGCCCATCTTC		ACGGGGGAGAGAGC
E3B2	CAGAAGACTCCCGCCCATCTT	GCCGGG	TACGGGGGAGAGAGC
E1F3	CAGAAGACTCCCGCCCATCTTC		GGGGGAGAGAGC
E1H12	CAGAAGACTCCCGCCC		GGATACGGGGGAGAGAGC
E3F7	CAGAAGACTCCCGCCCA		ATACGGGGGAGAGAGC
E1D4	CAGAAGACTCCCGCC		GGATACGGGGGAGAGAGC
E1E9	CAGAAGACTCCCGCC		GGATACGGGGGAGAGAGC
E2F2	CAGAAGACTCCCGCC		GGATACGGGGGAGAGAGC
E2G5	CAGAAGACTCCCGCC		GGATACGGGGGAGAGAGC
E3C1	CAGAAGACTCCCGCC		GGATACGGGGGAGAGAGC
E3F11	CAGAAGACTCCCGCC		GGATACGGGGGAGAGAGC
E2C1	CAGAAGACTCCCG	TAT	CCGGATACGGGGGAGAGAGC
E2A12	CAGAAGACTCCC	C	CCGGATACGGGGGAGAGAGC
E3G12	CAGAAGACTCCCG		GGATACGGGGGAGAGAGC
E1B7	CAGAAGACTCCCG		GATACGGGGGAGAGAGC
E1H9	CAGAAGACTCCCG		GATACGGGGGAGAGAGC
E2C5	CAGAAGACTCCCG		GATACGGGGGAGAGAGC
E2D11	CAGAAGACTCCCG		GATACGGGGGAGAGAGC
E3E4	CAGAAGACTCCCG		GATACGGGGGAGAGAGC
E3F8	CAGAAGACTCC	+381 bp	GATACGGGGGAGAGAGC
E2B2	CAGAAGACTCCCGCC		GGGGGAGAGAGC
E2A9	CAGAAGACTCCCGCC		GGGGAGAGAGC
E1H3	CAGAAGACTCCCGC		GGGGGAGAGAGC
E2D12	CAGAAGACTCCC		GGGGAGAGAGC
E1E6	CAGAAGAC		GGGGGAGAGAGC
E2H5	CAGAAGAC		GGGGGAGAGAGC
E3B3	CAG		GGGAGAGAGC
E1C9	GCCT -25 del		GGGGGAGAGAGC
E1A4	CAGA		GAGAGC
E2H2	GCCT -25 del	+33 bp	GGGGGAGAGAGC
E1H10	CTGC -23 del		-25 del TTAAG
E3H12	CAG		-30 del TTGAA

Figure S5, con't.

G) *Lig3*$^{KO/KO}$; *Lig3 ΔBRCT GFP*

Translocation clones	Chr. 6<br>CAGAAGACTCCCGCCCATCTTC	Translocation junctions	Chr. 11<br>CTCGCCGGATACGGGGGGAGAGAGC
E1C6	GTTAG -36 del		GGGGAGAGAGC
E2A3	GCCTT -24 del		-27 del AAGTT
E3A9	CAGAAG		-36 del CGGTT
E2H6	TAGC -35 del		-25 del TTAAG
E1A12	TTTAAG -30 del		-30 del TTGAA
E1A5	GGTTA -37 del		-28 del AGTTG
E3D6	GCCTT -32 del	+39bp	-54 del TGTAG

Figure S5, con't.

H) Xrcc4−/−

Translocation clones	Chr. 6		Chr. 11
	CAGAAGACTCCCGCCCATCTTC		(G)CTCGCCGGATACGGGGGGAGAGAGC
		Translocation junctions	
E2F9	CAGAAGACTCCCGCCCATCTTC		CTCGCCGGATACGGGGGGAGAGAGC
E1C6	CAGAAGACTCCCGCCCATCTTC		GCCGGATACGGGGGGAGAGAGC
E2A1	CAGAAGACTCCCGCCCATCTTC		GCCGGATACGGGGGGAGAGAGC
E2B8	CAGAAGACTCCCGCCCATCTTC		GCCGGATACGGGGGGAGAGAGC
E2G5	CAGAAGACTCCCGCCCATCT		CGCCGGATACGGGGGGAGAGAGC
E2C10	CAGAAGACTCCCGCCCATCT		CGCCGGATACGGGGGGAGAGAGC
E2D5	CAGAAGACTCCCGCCCAT		GCTCGCCGGATACGGGGGGAGAGAGC
E1G10	CAGAAGACTCCCGCCCATCT		CGCCGGATACGGGGGGAGAGAGC
E2F1	CAGAAGACTCCCGCCC	C	GCTCGCCGGATACGGGGGGAGAGAGC
E2C8	CAGAAGACTCCCGCC		GCTCGCCGGATACGGGGGGAGAGAGC
E2E10	CAGAAGACTCCCGCCCATCT		GGATACGGGGGGAGAGAGC
E1F6	CAGAAGACTCCCGCCCATC		CGGATACGGGGGGAGAGAGC
E1F7	CAGAAGACTCCCG		GATACGGGGGGAGAGAGC
E1A12	CAGAAGACTCCCGCCC		CCGGATACGGGGGGAGAGAGC
E2F3	CAGAAGACTCCCGCCCAT		GGATACGGGGGGAGAGAGC
E1D4	CAGAAGACTCCCGCCCAT	TAT	GGATACGGGGGGAGAGAGC
E1A5	CAGAAGACTCCCGCC	GTGATCT	GCCGGATACGGGGGGAGAGAGC
E2C2	CAGAAGACTCCCGCC	GTGATCT	GCCGGATACGGGGGGAGAGAGC
E1F8	CAGAAGACTCCCGCCC		CGGATACGGGGGGAGAGAGC
E2E1	CAGAAGACTCCCGCCC		CGGATACGGGGGGAGAGAGC
E2D6	CAGAAGACTCCCGCCCATCT		ACGGGGGGAGAGAGC
E1B2	CAGAAGACTCCCGCCCATC		TACGGGGGGAGAGAGC
E1B5	CAGAAGACTCCCGCCCA		GATACGGGGGGAGAGAGC
E1G12	CAGAAGACTCCCGCCCATCTTC		GGGGGGAGAGAGC
E1A2	CAGAAGACTCCCGCCC		GGATACGGGGGGAGAGAGC
E1E7	CAGAAGACTCCCGCCC		GGATACGGGGGGAGAGAGC
E2E3	CAGAAGACTCCCGCCC		GGATACGGGGGGAGAGAGC
E1F4	CAGAAGACTCCCGCC	+355 bp	CGGATACGGGGGGAGAGAGC
E2H7	CAGAAGACTCCCGCC	+213 bp	CGGATACGGGGGGAGAGAGC
E2B11	CAGAAGACTCCCGCC		GGATACGGGGGGAGAGAGC
E1F1	CAGAAGACTCCCGCC		GGATACGGGGGGAGAGAGC
E1G1	CAGAAGACTCCCGCC		GGATACGGGGGGAGAGAGC
E1A6	CAGAAGACTCCCGCC		GGATACGGGGGGAGAGAGC
E1D12	CAGAAGACTCCCGCC		GGATACGGGGGGAGAGAGC
E2F2	CAGAAGACTCCCGCC		GGATACGGGGGGAGAGAGC
E2A4	CAGAAGACTCCCGCC		GGATACGGGGGGAGAGAGC
E2A2	CAGAAGACTCCCGCCCATC		CGGGGGGAGAGAGC
E1H5	CAGAAGACTCCCGCCCAT		ACGGGGGGAGAGAGC
E1A11	CAGAAGACTCCCGC	ACG	GGATACGGGGGGAGAGAGC
E2H1	CAGAAGACTCCCGC	ATG	GGATACGGGGGGAGAGAGC
E2E9	CAGAAGACTCCCGC	A	GGATACGGGGGGAGAGAGC
E1D3	CAGAAGACTCCC		CGGATACGGGGGGAGAGAGC
E1H4	CAGAAGACTC		GCCGGATACGGGGGGAGAGAGC
E1A8	CAGAAGACTC		GCCGGATACGGGGGGAGAGAGC
E2G8	CAGAAGACTC		GCCGGATACGGGGGGAGAGAGC
E1A3	CAGAAGACTCCCG		GATACGGGGGGAGAGAGC
E1E8	CAGAAGACTCCCG		GATACGGGGGGAGAGAGC
E2C3	CAGAAGACTCCCG		GATACGGGGGGAGAGAGC
E2G6	CAGAAGACTCCCG		GATACGGGGGGAGAGAGC

Figure S5, con't.

H) Xrcc4−/−

Translocation clones	Chr. 6 CAGAAGACTCCCGCCCATCTTC	Translocation junctions	Chr. 11 (G)CTCGCCGGATACGGGGGGAGAGAGC
E1C4	CAGAAGACTCCC	CCC	GGATACGGGGGGAGAGAGC
E2E5	CAGAAGACTCCC		GATACGGGGGGAGAGAGC
E1F12	CAGAAGAC		GCCGGATACGGGGGGAGAGAGC
E1A4	CAGAAGACTC	CTGCCAGAAGACTC	CGGATACGGGGGGAGAGAGC
E1H11	CAGAAGACTCCCGCC	GGAGT	CGGGGGGAGAGAGC
E1C5	CAGAAGACTC		GATACGGGGGGAGAGAGC
E2G4	CAGAAGACTC		GCCGGATACGGGGGGAGAGAGC
E2G11	CAGAAGACTCCCGC		GGGAGAGAGC
E1H2	CAGAAGAC	CCCG	GATACGGGGGGAGAGAGC
E2G3	CAGAAGACTCCCG		GGGGGAGAGAGC
E2B1	CAGAAG		GATACGGGGGGAGAGAGC
E1C10	CAGAAGACT		ACGGGGGGAGAGAGC
E2A8	CAGAAGACTCCCGCCCA		GC
E2H4	CAGAA		GGGGGAGAGAGC
E2H2	CA	CC	ACGGGGGGAGAGAGC
E2D10	CAGA		GAGAGC
E2E7	TAGCC -31 del		GGATACGGGGGGAGAGAGC
E2B12	TAGCC -31 del		GGATACGGGGGGAGAGAGC
E2D7	CTTTA -27 del	GGATAC	GGGGGAGAGAGC
E2A7	CAG		GAGAGC
E1C9	AGCCT -33 del	+29 bp	CGGATACGGGGGGAGAGAGC
E1H12	CAG		AGAGC
E1D9	CAGA		GAGC
E1H3	CAGA		GC
E1B8	CAGAAGACTCCCG	AG	-35 del GCGGT
E1C2	CAGG -40 del		ATACGGGGGGAGAGAGC
E2A11	CACAC -46 del		CGGATACGGGGGGAGAGAGC
E1F3	CAGAAG		-37 del CGGTT
E2D8	CAGAAG		-37 del CGGGT
E2D12	CAGAAG		-37 del CGGGT
E1B4	TTAGC -35del	+136 bp	GAGC
E1E5	TAATA -208 del		CGGATACGGGGGGAGAGAGC

Figure S5, con't.

I) *Lig3*$^{KO/KO}$; *Lig3 GFP* + scrambled shRNA

Translocation clones	Chr. 6	Translocation junctions	Chr. 11
	CAGAAGACTCCCGCCCATCTTC		(G)CTCGCCGGATACGGGGGGAGAGAGC
E2A3	CAGAAGACTCCCGCCCATCTTC	T	TCGCCGGATACGGGGGGAGAGAGC
E1A7	CAGAAGACTCCCGCCCATCTTC		GCCGGATACGGGGGGAGAGAGC
E1G12	CAGAAGACTCCCGCCCATCTTC		GCCGGATACGGGGGGAGAGAGC
E1F8	CAGAAGACTCCCGCCCATCTTC	A	CGGATACGGGGGGAGAGAGC
E2E10	CAGAAGACTCCCGCCCAT	T	CTCGCCGGATACGGGGGGAGAGAGC
E2G11	CAGAAGACTCCCGCCCATCT		CCGGATACGGGGGGAGAGAGC
E2E12	CAGAAGACTCCCGCCC		CTCGCCGGATACGGGGGGAGAGAGC
E2D11	CAGAAGACTCCCGCCCATCTT		CGGATACGGGGGGAGAGAGC
E2H12	CAGAAGACTCCCGCCCATC		GGATACGGGGGGAGAGAGC
E1C9	CAGAAGACTCCCGCC	+716 bp	CCGGATACGGGGGGAGAGAGC
E1B5	CAGAAGACTCCCGCC		GGATACGGGGGGAGAGAGC
E2A2	CAGAAGACTCCCGCC		GGATACGGGGGGAGAGAGC
E2C9	CAGAAGACTCCCGCC		GGATACGGGGGGAGAGAGC
E2C10	CAGAAGACTCCCGCCCAT		ACGGGGGGAGAGAGC
E2E1	CAGAAGACTCCCGCCC	AGA	ATACGGGGGGAGAGAGC
E1F1	CAGAAGACTC		GCCGGATACGGGGGGAGAGAGC
E1G8	CAGAAGACTCCCG		GATACGGGGGGAGAGAGC
E1H12	CAGAAGACTCCCG		GATACGGGGGGAGAGAGC
E2A6	CAGAAGACTCCCG		GATACGGGGGGAGAGAGC
E1G3	CAGAAGACT		GGATACGGGGGGAGAGAGC
E2B11	CAGAAGAC	AT	CCGGATACGGGGGGAGAGAGC
E2F5	CAGAAGACTCCCG		GGGAGAGAGC
E2E6	CAGAAGA		TACGGGGGGAGAGAGC
E2B4	CAGAAGAC		GGGGGGAGAGAGC
E1H4	CAGAAG	G	ACGGGGGGAGAGAGC
E1C7	CAGA		TACGGGGGGAGAGAGC
E2A1	CAGAAGA		GGAGAGAGC
E1C11	CAGAAGACTCCCG	AG	GC
E1H6	CAGAAGACTCCCGC	ACG	-25 del TTAA[illegible]
E2E8	CAGAAGA		GAGC
E1F9	CAGAAGACTC		-26 del TAAG[illegible]
E1C6	CAGAAGA		-28 del GTTG[illegible]
E2A7	CAGAAG		-29 del TTGA[illegible]
E2E5	CAGAAG		-31 del CGGT[illegible]
E2C3	CAGGT -40 del		ACGGGGGGAGAGAGC
E2H9	CAGAAGAC		-110 del AATT[illegible]
E1E3	GCAGG -60 del	TGCACACCTG	-96 del TTGTA

Figure S5, con't.

J) *Lig3*$^{KO/KO}$; *Lig3 GFP* + Lig1 shRNA

Translocation clones	Chr. 6<br>CAGAAGACTCCCGCCCATCTTC	Translocation junctions	Chr. 11<br>(G)CTCGCCGGATACGGGGGAGAGAGC
E1H5	CAGAAGACTCCCGCCCATCTTC		CTCGCCGGATACGGGGGAGAGAGC
E2C1	CAGAAGACTCCCGCCCATCTT		GCTCGCCGGATACGGGGGAGAGAGC
E3B3	CAGAAGACTCCCGCCCATC		CTCGCCGGATACGGGGGAGAGAGC
E3E1	CAGAAGACTCCCGCCCATCTTC		GCCGGATACGGGGGAGAGAGC
E3B2	CAGAAGACTCCCGCCCATCTTC		GCCGGATACGGGGGAGAGAGC
E2H10	CAGAAGACTCCCGCCCATCT		CGCCGGATACGGGGGAGAGAGC
E3C2	CAGAAGACTCCCGCCCATCT		CGCCGGATACGGGGGAGAGAGC
E2F10	CAGAAGACTCCCGCCCAT		GCTCGCCGGATACGGGGGAGAGAGC
E2A6	CAGAAGACTCCCGCCCATC		CGCCGGATACGGGGGAGAGAGC
E3G5	CAGAAGACTCCCGCCCA		CTCGCCGGATACGGGGGAGAGAGC
E1C4	CAGAAGACTCCCGCCCATC		CGGATACGGGGGAGAGAGC
E3H1	CAGAAGACTCCCGCCCATCT		CCGGATACGGGGGAGAGAGC
E2B1	CAGAAGACTCCCGCCCAT		ACGGGGGAGAGAGC
E1D2	CAGAAGACTCCCGCCCAT		GCCGGATACGGGGGAGAGAGC
E3D10	CAGAAGACTCCCGCCCATC		CCGGATACGGGGGAGAGAGC
E2C10	CAGAAGACTCCCG		CTCGCCGGATACGGGGGAGAGAGC
E2D6	CAGAAGACTCCCGCCC		GGATACGGGGGAGAGAGC
E2G12	CAGAAGACTCCCGCCC		GGATACGGGGGAGAGAGC
E1A11	CAGAAGACTCCCG	T	GCCGGATACGGGGGAGAGAGC
E3E3	CAGAAGACTCCCGCCCATCTTC	+183 bp	GGGGGAGAGAGC
E1B7	CAGAAGACTCCCGCC		GGATACGGGGGAGAGAGC
E1H12	CAGAAGACTCCCGCC		GGATACGGGGGAGAGAGC
E2H1	CAGAAGACTCCCGCC		GGATACGGGGGAGAGAGC
E3F4	CAGAAGACTCCCGCC		GGATACGGGGGAGAGAGC
E3F7	CAGAAGACTCCCGCC		GGATACGGGGGAGAGAGC
E3E11	CAGAAGACTCCCGCC		GGATACGGGGGAGAGAGC
E2H4	CAGAAGACTCCCGCCCA		TACGGGGGAGAGAGC
E2E11	CAGAAGACTCCCGCCCA		TACGGGGGAGAGAGC
E1B3	CAGAAGACTCCCGCCCAT		ACGGGGGAGAGAGC
E3A9	CAGAAGACTCCCGCCCA		ACGGGGGAGAGAGC
E3E8	CAGAAGACTCCC		CGGATACGGGGGAGAGAGC
E1F5	CAGAAGACTC		GCCGGATACGGGGGAGAGAGC
E1E12	CAGAAGACTCCCG		GATACGGGGGAGAGAGC
E2A5	CAGAAGACTCCCG		GATACGGGGGAGAGAGC
E2E7	CAGAAGACTCCCG		GATACGGGGGAGAGAGC
E2A1	CAGAAGACTCCCGCCCA		CGGGGGAGAGAGC
E3C5	CAGAAGACTC		GCCGGATACGGGGGAGAGAGC
E3F10	CAGAAGACTCCCG		GGGAGAGAGC
E1A8	CAGAAGA		GAGAGC
E2B8	CAGAA		TACGGGGGAGAGAGC
E1A12	TTAAG -28 del	+48 bp	CGGATACGGGGGAGAGAGC
E1F10	CAGAA		GAGAGAGC
E1B4	CAGAAGACTCC		-25 del TTAA(
E1C2	CAGAA		GAGAGC
E1C7	CAGA		GC
E2H8	CAGA		GC

Figure S5, con't.

J) *Lig3*$^{KO/KO}$; *Lig3 GFP* + Lig1 shRNA

	Chr. 6		Chr. 11
	CAGAAGACTCCCGCCCATCTTC		(G)CTCGCCGGATACGGGGGGAGAGAGC
Translocation clones		Translocation junctions	
E3G12	CAGA		GC
E2A12	CAGAAG		-37 del CGGTT
E1C3	AGGTT -30 del	+55bp	-32 del GAAGC
E2D10	GCCTT -33 del		-32 del GAAGC
E3E2	CACCA -43 del	TAGCATTTAAGTGGGAAGATT	-25 del TTAAG
E1F9	CAGGA -59 del		GC
E1F1	CAGAAGACTCCCG		-89 del AAGTA
E3H9	CAGA	GTCTTCCATCGAGGTA	-161 del GATGA
E3B11	CCTAT -175 del		ACGGGGGGAGAGAGC
